# Theoretical Study of Metal–Ligand Interactions in Lead Complexes with Radiopharmaceutical Interest †

**DOI:** 10.3390/molecules29174198

**Published:** 2024-09-04

**Authors:** Attila Kovács, Zoltán Varga

**Affiliations:** 1 European Commission, Joint Research Centre (JRC), 76125 Karlsruhe, Germany; 2 Department of Chemistry, Chemical Theory Center, Minnesota Supercomputer Institute, University of Minnesota, Minneapolis, MN 55455, USA; zvarga@umn.edu

**Keywords:** radiopharmaceuticals, lead, chelating ligands, bonding, charge transfer, DFT

## Abstract

The ^203^Pb and ^212^Pb lead radioisotopes are attracting growing interest as they can aid in the development of personalized, targeted radionuclide treatment for advanced and currently untreatable cancers. In the present study, the bonding interactions of Pb^2+^ with twelve macrocyclic ligands, having an octa and nona coordination, were assessed using Density Functional Theory (DFT) calculations. The molecular structures in an aqueous solution were computed utilizing the polarized continuum model. The preference for the twisted square antiprismatic (TSAP) structure was confirmed for ten out of the eleven cyclen-based complexes. The characteristics of the bonding were assessed using a Natural Energy Decomposition Analysis (NEDA). The analysis revealed a strong electrostatic character of the bonding in the complexes, with minor variations in electrical terms. The charge transfer (CT) had a comparable energetic contribution only in the case of neutral ligands, while in general, it showed notable variations regarding the various donor groups. Our data confirmed the general superiority of the carboxylate O and aromatic N donors. The combination of the selected efficient pendant arms pointed out the superiority of the acetate pendant arms and the lack of significant cooperation between the different pendant arms in the probed ligands. Altogether, the combination led only to a marginal enhancement in the total CTs in the complexes.

## 1. Introduction

The lead radioisotopes ^203^Pb and ^212^Pb have recently received growing attention in radiopharmacy. The γ-emitting ^203^Pb isotope (279 keV, t_1/2_ = 51.9 h) is suitable for single-photon emission computed tomography (SPECT) imaging [1]. More interesting is the ^212^Pb isotope, which acts as an α-emitter generator by producing ^212^Bi (α, t_1/2_ = 61 min) through its β-decay. The relatively long half-life of ^212^Pb (β, t_1/2_ = 10.64 h) [2] can efficiently prolong the presence of the short-lived ^212^Bi in vivo, where the generated ^212^Bi isotope should effectively be complexed by the same chelator because its release and subsequent accumulation in off-target sites can lead to toxicity. Altogether, the two Pb radioisotopes form an ideal theranostic pair for radioimaging and targeted cancer radiotherapy [3,4,5] and are currently undergoing clinical trials.

In the above-mentioned pharmaceutical applications, the radionuclides must safely be driven to the biological target in order to avoid the radioactive contamination of other organs, which would lead to toxicity. This targeted delivery (and retention of the radionuclides) is usually performed in the form of chelate complexes, where the ligand is conjugated to a tumor-seeking vector. A strict requirement for these chelate complexes is that they should be thermodynamically highly stable and kinetically inert under in vivo conditions [3,4,6]. Accordingly, the design of appropriate chelators requires an understanding of the relationship between the chelator structure and the stability of the complex.

The main complexing characteristics of Pb^2+^ are the following: it is a borderline Lewis acid in terms of Hard and Soft Acid and Base (HSAB) theory [7,8,9], and in a solid state, it exhibits various coordinations, from mono- to dodecadentate coordinations, with oxygen, nitrogen, and thiolate groups [10]. Its effective ionic radius in eight-coordinate complexes is 1.29 Å [11], due to which Pb^2+^ belongs to the largest metal ions. Because its 6d valance orbitals are high in energy, the donor–acceptor interactions are managed by the partially occupied 6p orbitals. In addition, in some complexes, stereochemical activity was attributed to the occupied 6s orbital [12,13].

In terms of radiopharmaceutical research, several traditional and new ligands have been probed for Pb^2+^. The research includes experimental studies (X-ray crystallography, NMR spectroscopy, potentiometry, radiolabeling, challenge studies with other ligands—mostly EDTA—and metal ions under various solution conditions) [12,14,15,16,17,18,19,20,21,22,23,24,25,26,27,28] as well as a few quantum chemical calculations [12,13,20].

In particular, DFT calculations have been used to study the stereochemical activity of the occupied 6s orbital of Pb^2+^ [12,13], pointing out the importance of an asymmetric environment around Pb^2+^ for this feature. In addition, in a few cases, experimental studies have been assisted by DFT calculations on the structural (conformational) properties [20,29] of radiopharmaceutically relevant Pb^2+^ complexes. To the best of our knowledge, no systematic bonding analysis on such complexes has been reported so far.

**Figure 1:**
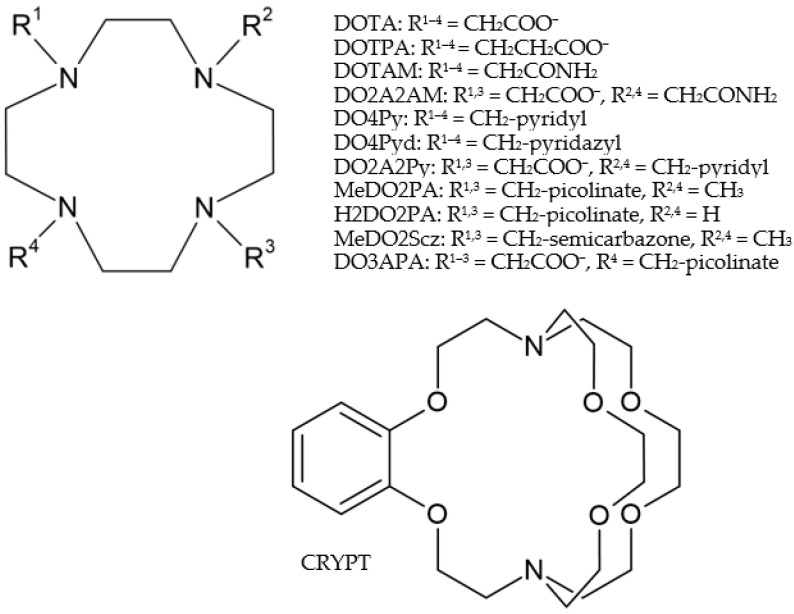
(**Top**) Compilation of the eleven cyclen-based ligands (the structure of each ligand is given in Figure S1 of the Supplementary Materials); (**Bottom**) Benzo substituted [2]-cryptand.

In the present study, we selected eleven macrocyclic cyclen-based ligands containing neutral and anionic pendant arms (cf. Figure 1 for notations used in the paper). Some of them have already been probed with Pb^2+^ (DOTA [15,25], with the current industry standard being DOTAM [16], DO4Py [26], MeDO2PA [20], H2DO2PA [20], and MeDO2Scz [21]). Two other ligands—showing good performance for Bi^3+^ and Ac^3+^ [30]—have promising structural properties: DOTPA has extended flexibility due to the longer pendant arms, and DO4Pyd has a modified charge and steric conditions at the aromatic N donor. The ligands with mixed pendant arms (DO2A2AM and DO2A2Py) are well suited to study the competition of two efficient donor groups in the same complex. With DO3APA, where one COO^−^ moiety of DOTA is replaced by a picolinate group, the possibility of nona-coordination with the large Pb^2+^ ion can be probed. Finally, the twelfth ligand in our comparative analysis is the benzo-substituted [2]-cryptand (CRYPT) macrocycle, which already has some experimental history with Pb^2+^ [23].

The ligand set presented above promises a versatile assessment of metal–ligand interactions. The Natural Energy Decomposition Analysis (NEDA) [31,32] provides, beyond the net interaction energies, details on the electrostatic, steric, and orbital interaction contributions. The efficiency of the individual donors in the donor–acceptor interactions can be deduced from the second-order perturbation energies obtained from the Natural Bond Orbital (NBO) [33] analysis. The information obtained can help design new efficient ligands for Pb radiopharmaceutical.

## 2. Results and Discussion

### 2.1. Structures

Except for CRYPT, the structures of the probed ligands are based on the twelve-membered tetraazamacrocycle cyclen and contain mostly four, while in some cases, two pendant arms (cf. Figure 1). The most stable structures of the complexes with ligands having four identical pendant arms (DOTA, DOTPA, DOTAM, DO4Py, and DO4Pyd) possess *C*_4_ symmetry, confirmed by our present calculations for aqueous solutions. The most stable structures of the complexes with ligands having two identical pendant arms (DO2A2AM, DO2A2Py, MeDO2PA, H2DO2PA, and MeDO2Scz) converged to minima with *C*_2_ symmetry on the potential energy surface. Two complexes in the set, those formed with ligands DO3APA and CRYPT, have *C*_1_ symmetry.

**Figure 2:**
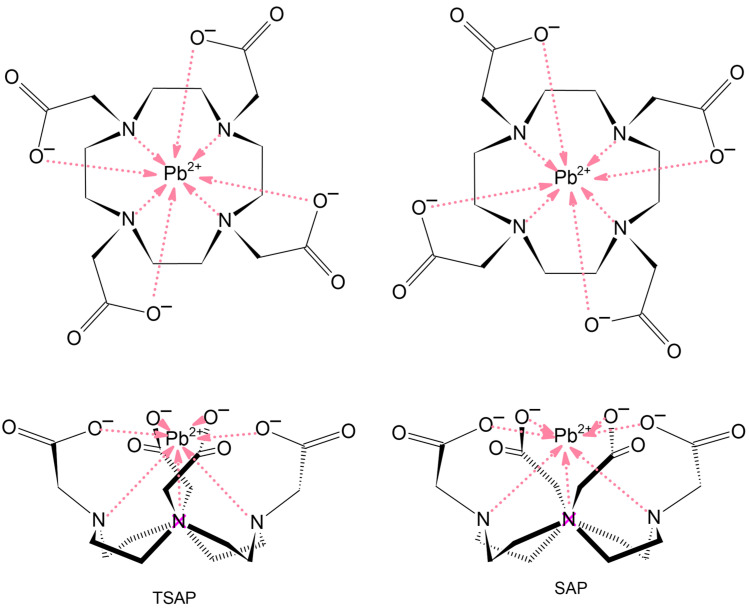
Schematic presentation of the two significant helical arrangements of (top) the pendant arms and (bottom) cyclen ring for the Pb(DOTA)^2−^ complex. The pink arrows indicate metal-ligand interactions.

The flexibility of the cyclen-based ligands leads to characteristic stereochemical properties of the complexes [34]. The ethylene groups in the cyclen macrocycle can take up δ- or λ-gauche orientations. The binding of the metal to the four N atoms fixes these to have four identical torsionals: δδδδ or λλλλ, respectively. The rotation of the acetate groups is limited upon coordination with the metal but still allows two opposite helical arrangements, Δ and Λ (Figure 2). The structures with the same helicities of the ring and the acetate groups, Δ(δδδδ) and Λ(λλλλ), respectively, are mirror images. Similarly, the forms with opposite helicities, Λ(δδδδ) and Δ(λλλλ), are enantiomers, too. The structure of the former conformers can be characterized as twisted square antiprismatic, leading to the conventional designation TSAP (or, in some literature, m). The latter conformers have a square antiprismatic coordination geometry (designated as SAP or M in the literature).

As the inversion barrier is rather low, the above enantiomers often appear as racemic mixtures in solutions [19,20,25] (and consequently upon crystallization from solutions [19,20,21]). The structures with the same ring and acetate helicity vs. the ones with opposite helicity are diastereomeric pairs and possess somewhat different spatial properties. The consequence is size selectivity: large metals usually form complexes with the TSAP conformer of these ligands, whereas small metals prefer the SAP conformer [35].

Our computations on the Pb complexes resulted in the energetic preference of the TSAP conformer in an aqueous solution for almost all complexes (cf. Table 1), in agreement with experimental reports for the solid-state structures of some of them [12,18,19,20,21,23,25,26]. The only exception was the Pb(DOTPA)^2−^ complex with a significant energetic preference for the SAP isomer. The DOTPA ligand differs from the other ten cyclen-based ones by its longer pendant arms, forming six-membered chelate rings with the metal. This can facilitate more favorable interactions with large metals in the SAP structures than in the cases of the other ligands forming five-membered chelate rings. It is noteworthy that the TSAP isomers of the ten other cyclen-based complexes are markedly more stable than the SAP ones. The energetic preference dropped below 10 kJ/mol only for the complexes with dipicolinate ligands (MeDO2PA, H2DO2PA, cf. Table 1).

Another characteristic of the cyclen-based complexes with large metals is the possible additional coordination of a small ligand (solvent, counterion, etc.) at a ninth coordination site. For example, in the case of the best-known DOTA with large metal ions (early lanthanides and actinides), such coordination has often been observed in the solid phase [36,37,38,39,40,41,42,43].

As Pb^2+^ belongs to the largest metal ions (vide supra), we probed the coordination of an H_2_O molecule at the ninth coordination site of the Pb(DOTA)^2−^ complex in an aqueous solution using the PCM solvation model. First, we constrained the H_2_O molecule in the middle of the free site; this model structure had a *C*_2_ symmetry. The geometry optimization resulted in a rather long Pb-O_H2O_ distance (3.3 Å). Moreover, this structure proved to be a third-order saddle-point on the potential energy surface. Lifting the symmetry constraint, the H_2_O ligand moved closer to two neighboring pendant arms and established very strong hydrogen bonds with the two carboxylate groups. In this minimum structure, the oxygen of H_2_O turned away from Pb^2+^, resulting in a Pb-O_H2O_ distance of 3.6 Å, which prevented any reasonable Pb^2+^-H_2_O interaction. Thus, our solvation model calculations do not support bonding of H_2_O to Pb^2+^ in the Pb(DOTA)^2+^ complex due to the stronger competitive effects of hydrogen bonding and outwards stabilization of the H_2_O oxygen by the polar solvent. Based on this experience, we skipped the study of additional H_2_O coordination for the other complexes.

**Figure 3:**
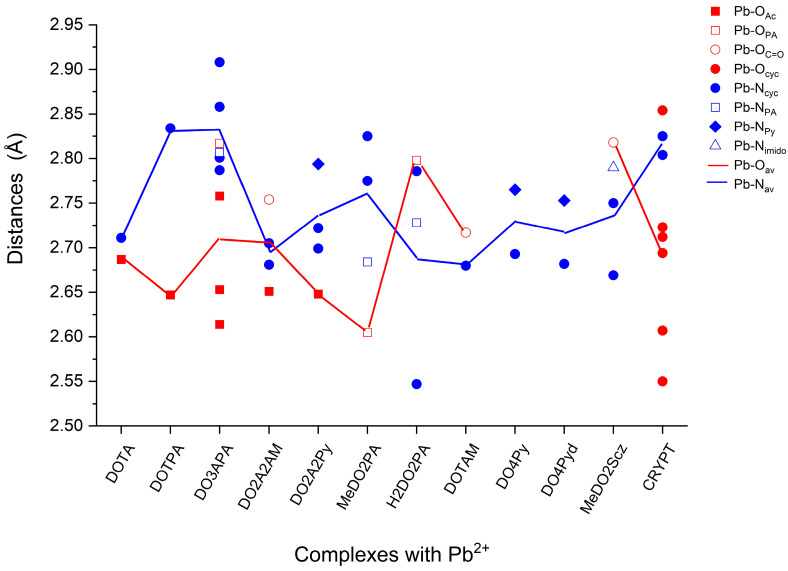
Individual donor–acceptor (symbols) and the average Pb-O and Pb-N distances (straight lines) in the complexes. The depicted values are given in Table 1 and Table S1.

Selected structural characteristics are given in Table 1 and Figure 3. We note that comparison of the Pb-O and Pb-N distances with most available X-ray diffraction values is not straightforward: the related crystal structures suffer from intermolecular interactions with counterions and solvent species [12,18,20,21,23] and/or dimer formation [19,21]. The only exception found is the crystal of [PbDO4Py](ClO_4_)_2_, in which the complex moiety has only marginal interactions with the ClO_4_^−^ counterions due to the efficient encapsulation of the metal (the closest ClO_4_^−^ has a Pb-O contact of 4.55 Å [26]). In addition, the donor N atoms of the pyridyl pendant arms are well shielded against intermolecular interactions. Yet, solid-state effects introduce asymmetry in the molecular structure in the crystal with variations up to 0.11 Å for the four Pb-N_Py_ distances, which are equivalent in the computed structure with *C*_4_ symmetry (cf. Table 1). Considering the different conditions, viz., the solution from the calculations while solid phase from the experiment, the average Pb-N distances from the X-ray study and our computations are in reasonably good agreement: 2.641(2) and 2.773(3) [26] vs. 2.693 and 2.765 Å for the cyclen and pyridyl N-s, respectively.

The exchange of pendant arms resulted in several significant changes in the Pb-O and Pb-N bond distances. These also appear in the bonding interactions (vide infra) because the electrostatic interactions are strongly distance-dependent, similar to the overlap of donor and acceptor orbitals for charge transfer.

Due to the *C*_4_ symmetry of Pb(DOTPA)^2−^ and Pb(DOTA)^2−^, Figure 3 clearly demonstrates how the propionate arms in DOTPA pulled the Pb^2+^ ion out from the cavity of the cyclene ring compared to the parent DOTA complex. The Pb^2+^ ion readily followed the lengthening of the pendant arm, proving the much stronger character of the carboxylate groups with respect to the cyclen N-s.

The picolinate pendant arm in the DO3APA ligand caused a significant distortion with respect to the *C*_4_ parent Pb(DOTA)^2−^ complex. Apparently, due to the larger picolinate group, the space around Pb^2+^ is more crowded in this complex, which resulted in an increase for most metal–ligand distances as a consequence of the nona-coordination. The increased distances imply a weakening of those metal–ligand interactions. On the other hand, the distortion resulted in the shortening of two Pb-O_Ac_ distances, which increased interactions could provide certain compensation for the weakening. Despite their good donor properties, the O and N donors of the picolinate group were positioned relatively far from Pb^2+^, probably due to steric effects.

Replacement of two acetate O donors of DOTA by neutral O and N in DO2A2AM and DO2A2Py, respectively, decreased both the Pb-O_Ac_ and Pb-N_cyc_ distances, implying a strengthening of these interactions. On the other hand, the distances with the neutral O and N donors were relatively large; they were significantly larger than in their parent DOTAM and DO4Py complexes. It seems that combining the efficient acetate, amide, and pyridyl donor groups did not lead to a synergic cooperation with significant enhancement in the bonding.

The ligands DO4Py and DO4Pyd differ by the meta-positioned N in the pyridazyl arms of the latter ligand. This N is not in an advanced position for bonding interactions with Pb^2+^ in the complexes, while it influences the charge density of the neighboring N and, consequently, those Pb-N interactions. Pyridazine is known to be less basic than pyridine due to the competitive electron-withdrawing of the two neighboring N-s. On this basis, weaker electrostatic and donor–acceptor interactions with Pb^2+^ would be expected. Yet, the Pb-N distances in Pb(DO4Pyd)^2+^ are slightly smaller than in Pb(DO4Py)^2+^. The explanation lies in the steric conditions: the CH hydrogen at the meta-position in the Py arm exerts a steric repulsion which limits the mutual proximity of the Py groups and, consequently, the access to Pb^2+^ in the Pb(DO4Py)^2+^ complex. The replacement of CH with N in Pb(DO4Pyd)^2+^ allowed a more efficient approach of the pendant arm to Pb^2+^.

The ligands MeDO2PA and H2DO2PA differ only by the presence of the methyl substituents on the cyclen backbone in the former molecule. Yet, the complex stabilities with Pb^2+^ are significantly different (log K_PbL_ = 18.44 ± 0.02 and 16.44 ± 0.02, respectively [20]), and similarly, so are the Pb-ligand distances in Figure 3: the Pb-O distances differ by 0.19 Å, whereas the Pb-N_cyc_ ones differ up to 0.28 Å. The major cause of such drastic differences cannot be explained by the steric effect because both the Me group and NH hydrogen have exo orientations on the cyclen backbone. However, this orientation facilitates NH to form a hydrogen bond with the carboxylate O (2.25 Å), which, in this way, is involved in a second interaction beyond the Pb-O one. The increase in the Pb-O distance by 0.19 Å, with respect to that in the Pb(MeDO2PA) complex, suggests the superiority of the hydrogen bonding. Also, the N donor in the picolinate group has an increased distance to Pb^2+^ (though only by 0.05 Å) in the Pb(H2DO2PA) complex. In parallel, the NH nitrogen of the cyclen backbone comes very close to Pb^2+^ (2.55 Å, representing the smallest Pb-N distance in the present set, cf. Figure 3), probably due to the increased polarized character of the NH group upon hydrogen bonding.

Replacing the strong anionic picolinate donor of MeDO2PA with the neutral one in DO2Scz resulted in the expected weakening of the Pb-O and, consequently, some strengthening of the competitive Pb-N_cyc_ interactions in the Pb(DO2Scz)^2+^ complex with respect to Pb(MeDO2PA). Coordination of the imido N to Pb^2+^ proved also to be considerably weaker than that of the picolinate N.

The coordination of the asymmetric CRYPT ligand has one noteworthy structural feature: the Pb(CRYPT)^2+^ complex has the shortest Pb-O distances (2.55 and 2.61 Å) in the present set formed by the two O-s of the middle bridge. Regarding these O-s being formally neutral donors, the main reason may lie in the spatial conditions that regulate the arrangement of the donor heteroatoms in this macrocycle ligand.

### 2.2. Bonding Analysis

The energetic properties of metal–ligand interactions were explored with Natural Energy Decomposition Analysis (NEDA) [31,32], which is an energy partitioning approach for molecular interactions applicable to self-consistent field (SCF) wavefunctions and DFT charge densities. The total interaction energy (ΔE_INT_) between the appropriately selected fragments consists of electrical interaction (EL), charge transfer (CT), and core repulsion (CORE) contributions according to:

ΔE_INT_ = ΔE_EL_ + ΔE_CT_ + ΔE_CORE_

**Figure 4:**
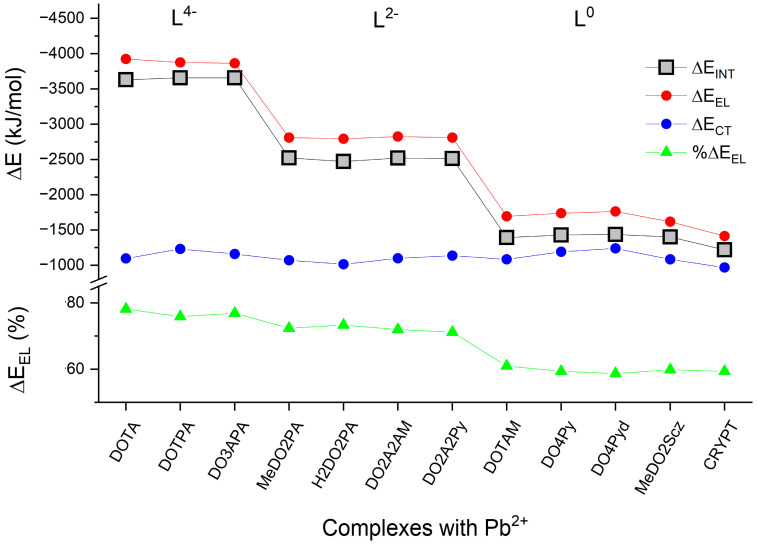
Results of the NEDA analysis grouped according the charges of the free ligands (L^4−^, L^2−^, L^0^). ΔE_EL_ (%) = 100·ΔE_EL_/(ΔE_EL_ + ΔE_CT_).

An overview of selected NEDA results is presented in Figure 4. While there is no direct relationship between the metal–ligand interaction energy and the complex stability in solution (the latter is strongly influenced by the solvation energy of the free ligand, which has a different structure than in the complex), it is remarkable that the very stable Pb(DOTA)^2+^ complex (experimental stability constant log K_PbL_ = 25.3 ± 0.2 [25]) has also one of the largest computed interaction energies. Experimental log K_PbL_ data are also available for Pb(DOTAM)^2+^ and Pb(DOPy4)^2+^ formed by neutral ligands (log K_PbL_ > 19 [16] and 19.95 ± 0.03 [26], respectively), indicating that a negatively charged ligand is not a prerequisite for complex stability in solution, as these complexes belong to those with the lowest metal–ligand interaction energies in the present set. In contrast, the Pb complexes with the negatively (2−) charged MeDO2PA and H2DO2PA ligands have even lower stabilities in solution (log K_PbL_ determined to be 18.44 ± 0.02 and 16.44 ± 0.02, respectively [20]). Among the latter two complexes, the weaker character of Pb(H2DO2PA) is in line with its lower computed interaction energy.

An interesting question is the efficiency of the nona-coordination in Pb(DO3APA)^2−^: the ΔE_INT_ data suggest only a marginal improvement of the bonding, as the complex has interaction energies close to those of Pb(DOTA)^2−^ and Pb(DOTPA)^2−^. As discussed in the previous section, the additional coordination in Pb(DO3APA)^2−^ could only be facilitated by the cost of several increased metal–ligand distances (vide supra in Figure 3), thus weakening those interactions.

The data in Figure 4 demonstrate nicely the dominant role of the free ligand’s charge for the total interaction energies. These ligand charges determine the electrical interaction, which is the major bonding component between Pb^2+^ and the ligands in the complexes. The portion of ΔE_EL_ within the bonding ΔE_EL_ + ΔE_CT_ interactions is between 59 and 78%, the lowest values belonging obviously to the neutral L^0^ ligands.

In contrast, the covalent charge transfer interactions have close energies (within 200 kJ/mol cf. Figure 4) independently from the charge of the free ligand. Ligands with aromatic N-donor pendant arms (DO4Py, DO4Pyd, DO2A2Py) have somewhat larger CT energy compared with the complexes possessing O-donor pendant arms.

The stronger CT interaction in Pb(DOTPA)^2−^ with respect to Pb(DOTA)^2−^ can be attributed to the flexibility of the longer propionate pendant arm (better orbital overlaps) with respect to the acetate one. In contrast, the spatial conditions for the electrical interactions seem to be more favorable in Pb(DOTA)^2−^. Yet, this, together with the weaker repulsive core interaction (cf. Table 2), is not enough to compensate for the smaller CT.

The effect of the methyl substituent on the cyclen ring can be assessed by comparing Pb(MeDO2PA) and Pb(H2DO2PA). Both the EL and CT bonding contributions are larger in Pb(MeDO2PA), while the slightly larger core repulsion does not compensate for it; hence, the methyl substitution results in a slightly larger interaction energy in Pb(MeDO2PA). Similar observations can be made for the related DO4Py and DO4Pyd complexes with a slight preference for Pb(DO4Pyd)^2+^.

The energetic contribution of the various donor atoms to the ΔE_CT_ term can be distinguished based on the second-order perturbation energies from Natural Bond Orbital (NBO) analysis [33]. In Table 2, the summed contributions of all the O and N donors, respectively, are shown. The data demonstrate the dominance of the carboxylate O donors in the cases with four COO-type pendant arms. The donation efficiency of the amide C=O groups is weaker, shown by its drop below that of the cyclen N-s in Pb(DOTAM)^2+^. Substitution of a carboxylate group by picolinate in Pb(DO3APA)^2−^ decreased slightly the summed O-donation with respect to Pb(DOTA)^2−^, while the additional fifth (picolinate) N donor increased significantly the summed N-donation. Altogether, the nona-coordination in Pb(DO3APA)^2−^ resulted in more favorable CT interactions with respect to the parent Pb(DOTA)^2−^. The increase in the pendant arms in Pb(DOTPA)^2−^ has a similar consequence.

The effect of the methyl substituent in Pb(MeDO2PA) vs. Pb(H2DO2PA) is the significant increase in the O-donation efficiency for the cost of the N-donation-in agreement with the changes in the Pb-O and Pb-N distances (cf. Figure 3). The N-donation is very efficient in complexes with pyridyl-type ligands (Py, Pyd), exceeding the carboxylate O of DOTA. This efficiency appears also in the mixed Pb(DO2A2Py) complex.

The semicarbazone pendant arms with C=N and C=O donor groups in Pb(MeDO2Scz)^2+^ show medium CT efficiency, while the ring O and N donors of the cryptand macrocycle in Pb(CRYPT)^2+^ exert the weakest CT interactions with Pb^2+^ in the present set of ligands.

The transferred number of electrons in the 12 complexes show relatively small variations (between 0.46 and 0.56 e, cf. Table 2), the trend being more-or-less in qualitative agreement with the CT energies.

**Figure 5:**
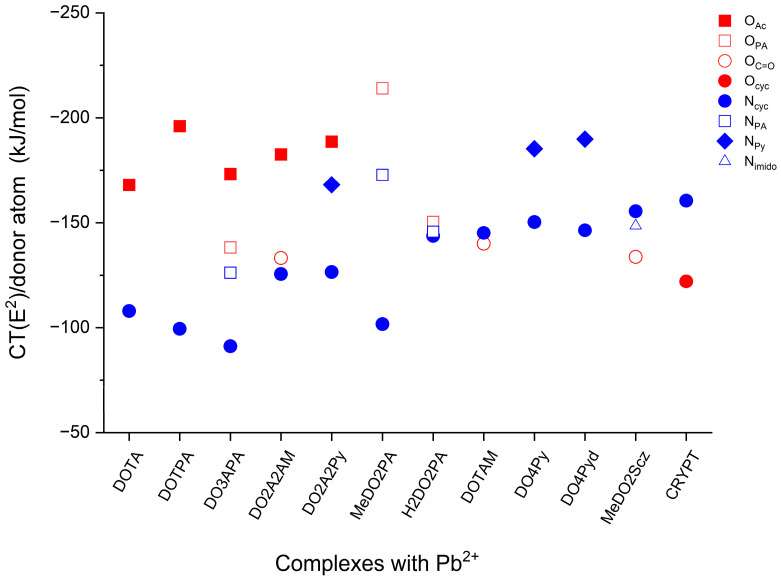
Averaged CT energies (kJ/mol) for the various donor types from the second-order perturbation energies (E^2^). The depicted values are given in Table S2.

The CT efficiency of the individual O and N donors can be followed in Figure 5. The superiority of the acetate/propionate O and aromatic N among these donors is evident. In most ligands, the weakest donors are the cyclen N-s. Exceptions are DOTAM and MeDO2Scz, where they outperform the amide C=O groups and, in the case of MeDO2Scz, also the imido C=N group. In the Pb(CRYPT)^2+^ complex, the tertiary N outperforms the secondary O donors.

Figure 5 also provides detailed information on the consequences of the probed exchanges of pendant arms. Thus, the replacement of one or more COO^−^ groups of DOTA with CH_2_COO^−^ (DOTPA), picolinate (DO3APA), amide (DO2A2AM), and pyridyl groups (DO2A2Py) increased the donation efficiency of the carboxylate O in these complexes in agreement with these decreased Pb-O_Ac_ distances (vide supra Figure 3). In contrast, the O and N donors in most of the above-added groups perform generally weaker in terms of CT than in their parent Pb(DOTAM)^2+^ and Pb(DO4Py)^2+^ or related Pb(MeDO2PA) complexes.

It is noteworthy that the replacement of the methyl groups in the MeDO2PA ligand with hydrogens in H2DO2PA (and the above-discussed hydrogen bonding) more-or-less equalized the CT from the three donor types, whereas these CT-s in the Pb(MeDO2PA) complex differ significantly.

Another enlightening feature appears in the DO4Py vs. DO4Pyd complexes: the decreased Pb-N_cyc_ and Pb-N_Py_ distances in Pb(DO4Pyd)^2+^ result only in the case of N_Py_ increased CT energy; the CT performance of the N_cyc_ donors became slightly weaker.

The natural atomic charges and valence orbital populations of Pb are given in Table S3. The latter data agree with previous findings that the 6p orbitals are the major acceptors in Pb^2+^. Accordingly, their natural population varies between 0.47 and 0.56 e, following near quantitively the transferred electrons from the ligands. The 6s populations change only marginally, while the 6d natural populations are negligible.

## 3. Methods

The DFT calculations were carried out with the Gaussian09 suite of programs [44] using the PBE0 exchange-correlation functional [45,46]. This function was selected on the basis of its very good general performance [47,48], particularly for metal complexes [48,49]; therefore, it is applied frequently in such studies [29,50]. For Pb, the small-core relativistic Stuttgart–Cologne pseudopotential (ECP60MDF) [51] in conjunction with a contracted (12s11p8d1f)/[5s4p3d1f] basis set of triple-zeta plus polarization quality [52], whereas for the light atoms the standard 6-311G** basis set was used. The latter bases of O and N were extended by one set of diffuse functions. The dispersion forces were taken into account with the D3 version of Grimme’s dispersion correction [53], applying Becke–Johnson damping [54]. The SuperFine grid was applied for good integration accuracy. It contained 175 radial shells and 974 angular points (175,974) per shell for H, C, O, and N, whereas for Pb, it was 250,974.

In the geometry optimizations the aqueous solution conditions were taken into account by means of the polarizable continuum model (PCM) [55,56]. The initial structures for geometry optimizations of the complexes were taken from the literature when available. In the case of computed literature structures, we chose those of the related Ac complexes based on the similar ionic radii of Pb^2+^ and Ac^3+^ [11]: they were the DOTA, DOTPA, DO4Py, DO4Pyd, and MeDO2PA complexes from Ref. [30]. Reported crystal structures of the Pb complexes were used for the DOTAM [19], CRYPT [23], and semicarbazone complexes [21]. The initial structures of the H2DO2PA, DO2A2AM, DO2A2Py, and DO3APA complexes were prepared manually from the optimized structures of the parent complexes. As the geometry optimizations often resulted in structures with *C*_1_ symmetry (either because of the crystal initial structures or due to symmetry break during the run), the resulting structures were inspected for possible symmetries and corrected if suited. For analysis of the geometries, the GaussView 5 software [57] was used. The minimum characters of all the optimized structures were confirmed by frequency analyses.

The natural atomic charges and valence orbital populations of Pb, as well as the second-order perturbation energies, were evaluated on the basis of the Natural Bond Orbital (NBO) model [33]. The metal–ligand interactions were further explored with Natural Energy Decomposition Analysis (NEDA) [31,32] using the NBO 7.0 code [58] coupled with Gaussian16 [59].

## 4. Conclusions

In the present DFT study, the structure and bonding characteristics of twelve complexes of the pharmaceutically important Pb^2+^ ion with macrocyclic ligands were assessed in an aqueous solution. The bonding analysis was based on NEDA as well as on second-order perturbation energies of the Fock matrix from the NBO model. The obtained computed results facilitated a differentiation between the various pendant arms containing O and N donors for the CT interactions.

With ten out of the eleven cyclen-based ligands, the TSAP conformers proved to be the most stable structures of the Pb complexes. Only the complex with the DOTPA ligand containing extended pendant arms favored the SAP isomer.

The total interaction energies from the NEDA analysis are comparable for the complexes with the same charge. In agreement with the anionic and/or strongly polarized character of the probed ligands, their magnitudes are determined by the electrical term. Its bonding contribution amounts to 59–78% of the total bonding energy (neutral ligands providing the smallest values). The different pendant arms of DOTPA and DO3APA increased marginally the total interaction energy with respect to that of the reference DOTA complex.

Whereas the electrical term depends strongly on the charge of the complex, it varies generally marginally among the complexes with the same charge. In contrast, the CT energies do not show any systematic relation to the charge of the complex but vary more than the EL among the complexes with the same charge. The energetic analysis revealed the CT superiority of the carboxylate O-s. From the N-s, the aromatic ones are generally the most efficient donors.

Combinations of selected efficient O- and N-donor pendant arms were probed in the DO3APA, DO2A2AM, and DO2A2Py complexes with reference to Pb(DOTA)^2−^—assessing only the CT interactions due to the different charges. While the replacement of one or more carboxylate groups of DOTA by the picolinate, amide, or pyridyl groups increased the donation efficiency of the remaining carboxylate groups, the new donor groups performed worse than in their parent complexes. This shows the superiority of the acetate pendant arms in such ligands and the lack of synergic cooperation between the donor arms. Altogether, the combination led only to marginal enhancement of the total CT in the complexes.

In general, carboxylate donor groups tend to form stronger bonds with Pb^2+^ than neutral ones, raising the question about a similar or better (?) efficiency of phosphonate and sulfonate groups. On the other hand, the change in the macrocycle moiety could make a significant difference in the bonding. In this direction, the recently reported diaza-18-crown-type [29,60] or bispidine-based ligands [61] may be worth studying.

## Figures and Tables

**Table 1:** Selected structural characteristics of the Pb complexes in aqueous solution ^1^.

Complex	ΔE_TSAP-SAP_	Pb-O_av_	Pb-N_av_	Symmetry
Pb(DOTA)^2−^	−22.8	2.687	2.711	*C*_4_
Pb(DOTPA)^2−^	43.7	2.647	2.834	*C*_4_
Pb(DOTAM)^2+^	−12.8	2.717	2.680	*C*_4_
Pb(DO2A2AM)	−18.9	2.702	2.693	*C*_2_
Pb(DO4Py)^2+^	−19.0	-	2.729	*C*_4_
Pb(DO4Pyd)^2+^	−25.1	-	2.718	*C*_4_
Pb(DO2A2Py)	−17.7	2.648	2.738	*C*_2_
Pb(MeDO2PA)	−8.5	2.605	2.761	*C*_2_
Pb(H2DO2PA)	−7.4	2.798	2.687	*C*_2_
Pb(MeDO2Scz)^2+^	−14.7	2.818	2.736	*C*_2_
Pb(DO3APA)^2−^	−18.6	2.711	2.832	*C*_1_
Pb(CRYPT)^2+^	-	2.690	2.815	*C*_1_

^1^ Energetic preference of the TSAP structures vs. the SAP ones in terms of electronic energy (kJ/mol); average Pb-O and Pb-N donor–acceptor distances (Å) of the most stable structures (TSAP except for the DOPTA complex); symmetry of the most stable structures.

**Table 2:** Selected NEDA results and additional NBO characteristics ^1^.

Complex			NEDA		CT(E^2^)		CT(e)
	ΔE_INT_	ΔE_EL_	ΔE_CORE_	ΔE_CT_	O → Pb^2+^	N → Pb^2+^	
Pb(DOTA)^2−^	−3630	−3924	1391	−1097	−672.1	−431.4	0.49
Pb(DOTPA)^2−^	−3657	−3877	1451	−1232	−784.1	−397.9	0.51
Pb(DO3APA)^2−^	−3657	−3863	1367	−1161	−658.0	−490.6	0.52
Pb(MeDO2PA)	−2523	−2810	1358	−1072	−428.0	−754.2	0.50
Pb(H2DO2PA)	−2471	−2794	1338	−1015	−300.7	−866.2	0.51
Pb(DO2A2AM)	−2520	−2825	1404	−1100	−631.4	−502.2	0.50
Pb(DO2A2Py)	−2513	−2810	1435	−1138	−377.1	−842.2	0.53
Pb(DOTAM)^2+^	−1394	−1694	1384	−1084	−559.8	−580.4	0.49
Pb(DO4Py)^2+^	−1429	−1739	1501	−1191	-	−1342.2	0.56
Pb(DO4Pyd)^2+^	−1438	−1763	1565	−1240	-	−1344.9	0.56
Pb(MeDO2Scz)^2+^	−1403	−1619	1302	−1086	−267.3	−919.0	0.54
Pb(CRYPT)^2+^	−1218	−1414	1164	−968	−731.9	−321.0	0.46

^1^ Energy data (kJ/mol) according to ΔE_INT_ = ΔE_EL_ + ΔE_CT_ + ΔE_CORE_. ΔE_INT_ means the total interaction energy between the two fragments Pb^2+^ and ligand consisting of electrical interaction (EL), charge transfer (CT), and core repulsion (CORE) contributions; summed energy consequences (kJ/mol) of the O → Pb^2+^ and N → Pb^2+^ charge transfers derived from the second-order perturbation energies between the ligand donor and Pb^2+^ acceptor orbitals; transferred number of electrons (e) from the ligands to Pb^2+^.

## Data Availability

All relevant data have been included in the paper and in the Supplementary Materials.

## Supplementary Materials

The following supporting information can be downloaded at https://www.mdpi.com/article/10.3390/molecules29174198/s1, Figure S1: 2D structures of the probed ligands; Table S1: Computed Pb-O and Pb-N distances; Table S2: CT energies for the various donor types; Table S3: Natural atomic charge and populations of the 6s, 6p and 6d orbitals of Pb in the complexes; Cartesian coordinates of the optimized TSAP and SAP structures.
