# Supplementary material for: Theoretical Study of Metal–Ligand Interactions in Lead Complexes with Radiopharmaceutical Interest
Source: Molecules. 2024 Sep 4;29(17):4198. doi: 10.3390/molecules29174198 (PMC11397547; doi:10.3390/molecules29174198)
Supplement: Supplementary file 1 [file molecules-29-04198-s001.zip › molecules-3103128-supplementary.pdf]

Supplementary Materials

Theoretical Study of Metal-Ligand Interactions in Lead Complexes with Radiopharmaceutical Interest

Attila Kovács [1,*] and Zoltán Varga [2]

[1] European Commission, Joint Research Centre (JRC), 76125 Karlsruhe, Germany
[2] Department of Chemistry, Chemical Theory Center, Minnesota Supercomputer Institute, University of Minnesota, Minneapolis, MN 55455, USA; zvarga@umn.edu
* Correspondence: attila.kovacs@ec.europa.eu

Figure S1. 2D structures of the probed ligands.

Table S1. Computed Pb-O and Pb-N distances.
Table S2. CT energies for the various donor types from the second-order perturbation energies (E^2).
Table S3. Natural atomic charge and populations of the 6s, 6p and 6d orbitals of Pb in the complexes.

Cartesian coordinates and PBE0 electronic energies of the optimized TSAP and SAP structures.

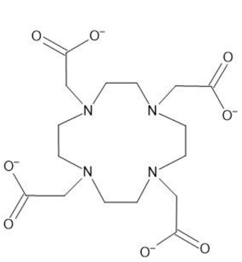

$DOTA^{4-}$

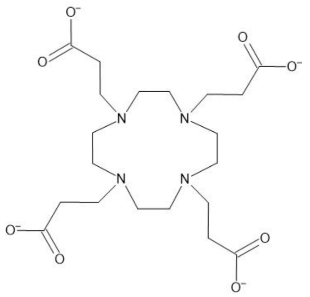

$DOTPA^{4-}$

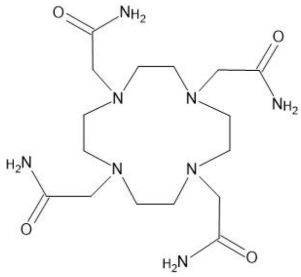

DOTAM

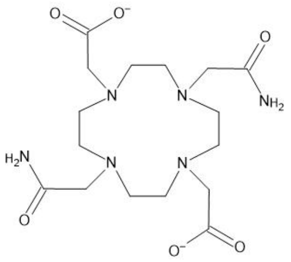

$DO2A2AM^{2-}$

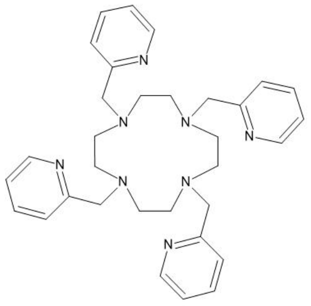

DO4Py

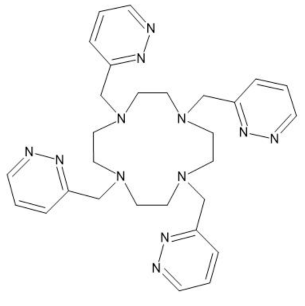

DO4Pyd

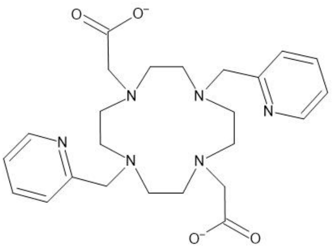

$DO2A2Py^{2-}$

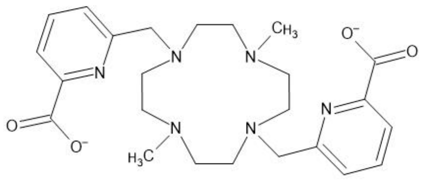

$MeDO2PA^{2-}$

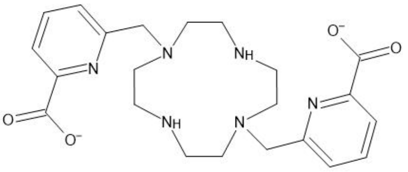

$H2DO2PA^{2-}$

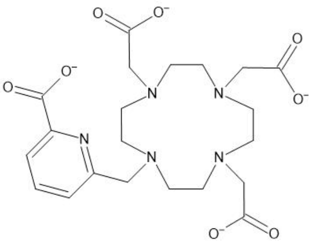

$DO3APA^{4-}$

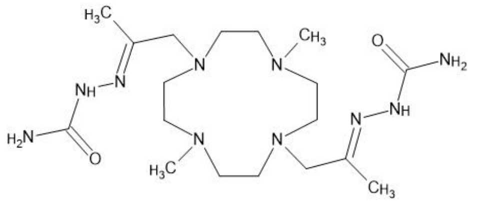

MeDO2Scz

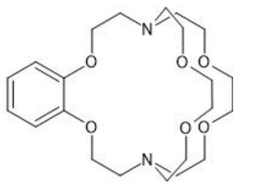

CRYPT

Figure S1. 2D structures of the probed ligands.

Table S1. Computed Pb-O and Pb-N distances (Å).

Ligand	Pb-O_{Ac}	Pb-O_{PA}	Pb-$O_{C=O}$	Pb-O_{cyc}	Pb-N_{cyc}	Pb-N_{cyc}	Pb-N_{PA}	Pb-N_{Py}	Pb-N_{imido}
DOTA	2.687				2.711	2.711			
DOTPA	2.647				2.834	2.834			
DO3APA	2.758	2.817			2.858	2.787	2.807		
	2.614				2.801	2.908			
	2.653								
DO2A2AM	2.651		2.754		2.681	2.705			
DO2A2Py	2.648				2.699	2.722		2.794	
MeDO2PA		2.605			2.825	2.775	2.684		
H2DO2PA		2.798			2.786	2.547	2.728		
DOTAM			2.717		2.680	2.680			
DO4Py					2.693	2.693		2.765	
DO4Pyd					2.682	2.682		2.753	
MeDO2Scz			2.818		2.669	2.750			2.790
CRYPT				2.694	2.825	2.804			
				2.712					
				2.723					
				2.854					
				2.550					
				2.607					

Table S2. CT energies for the various donor types from the second-order perturbation energies (E^2, kJ/mol).[a]

Ligand	O_{Ac}	O_{PA}	$O_{C=O}$	O_{cyc}	N_{cyc}	N_{PA}	N_{Py}	N_{imido}
DOTA	-168.0				-107.9			
DOTPA	-196.0				-99.5			
DO3APA	-173.2	-138.2			-91.1	-126.2		
DO2A2AM	-182.5		-133.2		-125.6			
DO2A2Py	-188.6				-126.5		-168.1	
MeDO2PA		-214.0			-101.7	-172.8		
H2DO2PA		-150.4			-143.7	-145.7		
DOTAM			-140.0		-145.1			
DO4Py					-150.3		-185.2	
DO4Pyd					-146.4		-189.8	
MeDO2Scz			-133.7		-155.5			-148.6
CRYPT				-122.0	-160.5			

[a]For non-equivalent groups (in the cases of lower symmetry) the values of the individual groups were averaged.

Table S3. Natural atomic charge and populations of the 6s, 6p and 6d orbitals of Pb in the complexes.

Ligand	q_{Pb}	6s	6p	6d
DOTA	1.51	1.98	0.49	0.01
DOTPA	1.49	1.98	0.51	0.01
DO3APA	1.48	1.98	0.52	0.00
MeDO2PA	1.5	1.98	0.51	0.01
H2DO2PA	1.49	1.96	0.54	0.01
DO2A2AM	1.50	1.98	0.51	0.01
DO2A2Py	1.47	1.98	0.53	0.02
DOTAM	1.51	1.98	0.49	0.01
DO4Py	1.44	1.97	0.56	0.02
DO4Pyd	1.44	1.96	0.56	0.02
MeDO2Scz	1.46	1.98	0.54	0.01
CRYPT	1.54	1.97	0.47	0.01

Cartesian coordinates and PBE0 electronic energies of the optimized TSAP and SAP structures.

Pb(DOTA)$^{2-}$ TSAP -1637.17064 Hartree

Pb	0.00000000	0.00000000	0.57190700
O	1.54115900	2.03457000	1.41097800
O	-2.03457000	1.54115900	1.41097800
O	-1.54115900	-2.03457000	1.41097800
O	2.03457000	-1.54115900	1.41097800
O	3.73250900	2.44440100	1.62280500
O	-2.44440100	3.73250900	1.62280500
O	-3.73250900	-2.44440100	1.62280500
O	2.44440100	-3.73250900	1.62280500
N	0.96469100	-1.88950200	-1.11625400
N	1.88950200	0.96469100	-1.11625400
N	-0.96469100	1.88950200	-1.11625400
N	-1.88950200	-0.96469100	-1.11625400
C	2.23693200	-1.43542100	-1.68389900
H	2.54263600	-2.08530000	-2.51884400
H	2.99863300	-1.53693200	-0.90996700
C	2.19813000	0.00000000	-2.17295900
H	3.16655900	0.23673900	-2.64149600
H	1.44859200	0.10464900	-2.96018600
C	1.43542100	2.23693200	-1.68389900
H	2.08530000	2.54263600	-2.51884400
H	1.53693200	2.99863300	-0.90996700
C	0.00000000	2.19813000	-2.17295900
H	-0.23673900	3.16655900	-2.64149600
H	-0.10464900	1.44859200	-2.96018600
C	-2.23693200	1.43542100	-1.68389900
H	-2.54263600	2.08530000	-2.51884400
H	-2.99863300	1.53693200	-0.90996700
C	-2.19813000	0.00000000	-2.17295900
H	-3.16655900	-0.23673900	-2.64149600
H	-1.44859200	-0.10464900	-2.96018600
C	-1.43542100	-2.23693200	-1.68389900
H	-2.08530000	-2.54263600	-2.51884400
H	-1.53693200	-2.99863300	-0.90996700
C	0.00000000	-2.19813000	-2.17295900
H	0.23673900	-3.16655900	-2.64149600
H	0.10464900	-1.44859200	-2.96018600
C	3.04564300	1.18413500	-0.25131100
H	3.85500600	1.69483300	-0.79413600
H	3.42813500	0.21337300	0.07512500
C	2.74293900	1.96613300	1.03632100
C	-1.18413500	3.04564300	-0.25131100
H	-1.69483300	3.85500600	-0.79413600
H	-0.21337300	3.42813500	0.07512500
C	-1.96613300	2.74293900	1.03632100
C	-3.04564300	-1.18413500	-0.25131100
H	-3.85500600	-1.69483300	-0.79413600
H	-3.42813500	-0.21337300	0.07512500
C	-2.74293900	-1.96613300	1.03632100
C	1.18413500	-3.04564300	-0.25131100
H	1.69483300	-3.85500600	-0.79413600
H	0.21337300	-3.42813500	0.07512500
C	1.96613300	-2.74293900	1.03632100

Pb(DOTA)$^{2-}$ SAP -1637.16195 Hartree

Pb	0.00000000	0.00000000	0.68991700
N	0.20246200	2.13733200	-1.01353600
N	2.13733200	-0.20246200	-1.01353600
N	-0.20246200	-2.13733200	-1.01353600
N	-2.13733200	0.20246200	-1.01353600
O	-1.39551900	2.17443200	1.37183200
O	2.17443200	1.39551900	1.37183200
O	1.39551900	-2.17443200	1.37183200
O	-2.17443200	-1.39551900	1.37183200
O	4.38814400	1.62779200	1.15344000
O	1.62779200	-4.38814400	1.15344000
O	-4.38814400	-1.62779200	1.15344000
O	-1.62779200	4.38814400	1.15344000
C	-1.12082700	3.28935600	0.85321400
C	3.28935600	1.12082700	0.85321400
C	1.12082700	-3.28935600	0.85321400
C	-3.28935600	-1.12082700	0.85321400
C	1.54863200	2.16254700	-1.59941400
C	2.03217700	0.80490500	-2.07161100
C	2.16254700	-1.54863200	-1.59941400
C	0.80490500	-2.03217700	-2.07161100
C	-1.54863200	-2.16254700	-1.59941400
C	-2.03217700	-0.80490500	-2.07161100
C	-2.16254700	1.54863200	-1.59941400
C	-0.80490500	2.03217700	-2.07161100
C	0.00000000	3.33530400	-0.19787900
C	3.33530400	0.00000000	-0.19787900
C	0.00000000	-3.33530400	-0.19787900
C	-3.33530400	0.00000000	-0.19787900
H	0.91506200	3.49076300	0.38184000
H	-0.14776300	4.21976900	-0.83402100
H	4.21976900	0.14776300	-0.83402100
H	3.49076300	-0.91506200	0.38184000
H	0.14776300	-4.21976900	-0.83402100
H	-0.91506200	-3.49076300	0.38184000
H	-3.49076300	0.91506200	0.38184000
H	-4.21976900	-0.14776300	-0.83402100
H	2.23175900	2.55392200	-0.84663400
H	1.58671800	2.85641300	-2.45416800
H	3.00947800	0.94052500	-2.56248300
H	1.35863700	0.42254000	-2.84110800
H	2.85641300	-1.58671800	-2.45416800
H	2.55392200	-2.23175900	-0.84663400
H	0.94052500	-3.00947800	-2.56248300
H	0.42254000	-1.35863700	-2.84110800
H	-1.58671800	-2.85641300	-2.45416800
H	-2.23175900	-2.55392200	-0.84663400
H	-1.35863700	-0.42254000	-2.84110800
H	-3.00947800	-0.94052500	-2.56248300
H	-2.55392200	2.23175900	-0.84663400
H	-2.85641300	1.58671800	-2.45416800
H	-0.42254000	1.35863700	-2.84110800
H	-0.94052500	3.00947800	-2.56248300

$Pb(DOTPA)^{2-}$ TSAP -1794.24276 Hartree

Pb	0.00000000	0.00000000	0.56656100
N	-0.20363600	2.21012800	-1.28711000
N	-2.21012800	-0.20363600	-1.28711000
N	2.21012800	0.20363600	-1.28711000
N	0.20363600	-2.21012800	-1.28711000
O	1.52810400	-1.87132400	1.69259500
O	-1.87132400	-1.52810400	1.69259500
O	-1.52810400	1.87132400	1.69259500
O	1.87132400	1.52810400	1.69259500
O	-3.77846200	-2.59739400	1.23678600
O	-2.59739400	3.77846200	1.23678600
O	3.77846200	2.59739400	1.23678600
O	2.59739400	-3.77846200	1.23678600
C	-3.07033300	-1.56864600	1.28154100
C	-2.14991500	-1.56796600	-1.83244000
C	-0.24603800	3.65836900	0.81042200
H	-0.21892200	4.72301800	1.06251000
H	0.56520400	3.14108100	1.32476500
C	0.79105800	2.00785400	-2.34321400
H	0.92904200	2.93628500	-2.92015300
H	0.41025300	1.27997500	-3.05855800
C	2.14991500	1.56796600	-1.83244000
H	2.87158500	1.66804000	-2.66127000
H	2.48401600	2.25039000	-1.04718100
H	-2.48401600	-2.25039000	-1.04718100
H	-2.87158500	-1.66804000	-2.66127000
C	-3.65836900	-0.24603800	0.81042200
H	-4.72301800	-0.21892200	1.06251000
H	-3.14108100	0.56520400	1.32476500
C	-2.00785400	0.79105800	-2.34321400
H	-2.93628500	0.92904200	-2.92015300
H	-1.27997500	0.41025300	-3.05855800
C	-1.56796600	2.14991500	-1.83244000
H	-1.66804000	2.87158500	-2.66127000
H	-2.25039000	2.48401600	-1.04718100
C	-1.56864600	3.07033300	1.28154100
H	-0.41025300	-1.27997500	-3.05855800
C	3.65836900	0.24603800	0.81042200
H	4.72301800	0.21892200	1.06251000
H	3.14108100	-0.56520400	1.32476500
C	2.00785400	-0.79105800	-2.34321400
H	2.93628500	-0.92904200	-2.92015300
H	1.27997500	-0.41025300	-3.05855800
C	1.56796600	-2.14991500	-1.83244000
H	1.66804000	-2.87158500	-2.66127000
H	2.25039000	-2.48401600	-1.04718100
H	-0.92904200	-2.93628500	-2.92015300
C	3.07033300	1.56864600	1.28154100
C	0.24603800	-3.65836900	0.81042200
H	0.21892200	-4.72301800	1.06251000
H	-0.56520400	-3.14108100	1.32476500
C	-0.79105800	-2.00785400	-2.34321400
C	1.56864600	-3.07033300	1.28154100
C	0.00000000	3.54849800	-0.68903600
H	-0.62099200	4.27273000	-1.23853200
H	1.03749600	3.84248000	-0.86498900
C	-3.54849800	0.00000000	-0.68903600
H	-4.27273000	-0.62099200	-1.23853200
H	-3.84248000	1.03749600	-0.86498900
C	0.00000000	-3.54849800	-0.68903600
H	0.62099200	-4.27273000	-1.23853200
H	-1.03749600	-3.84248000	-0.86498900
C	3.54849800	0.00000000	-0.68903600
H	4.27273000	0.62099200	-1.23853200
H	3.84248000	-1.03749600	-0.86498900

$Pb(DOTPA)^{2-}$ SAP -1794.25941 Hartree

Pb	0.00000000	0.00000000	0.30714600
N	-0.19610900	2.17442600	-1.49976300
N	2.17442600	0.19610900	-1.49976300
N	0.19610900	-2.17442600	-1.49976300
N	-2.17442600	-0.19610900	-1.49976300
O	1.31243900	-1.94577800	1.53043500
O	-1.94577800	-1.31243900	1.53043500
O	-1.31243900	1.94577800	1.53043500
O	1.94577800	1.31243900	1.53043500
O	3.66784600	0.69253500	2.81135700
O	-0.69253500	3.66784600	2.81135700
O	-3.66784600	-0.69253500	2.81135700
O	0.69253500	-3.66784600	2.81135700
C	0.00000000	3.67050200	0.53462700
C	3.67050200	0.00000000	0.53462700
C	0.00000000	-3.67050200	0.53462700
C	-3.67050200	0.00000000	0.53462700
C	1.13632800	2.40389500	-2.06330800
C	1.83861000	1.15232900	-2.55685400
C	2.40389500	-1.13632800	-2.06330800
C	1.15232900	-1.83861000	-2.55685400
C	-1.13632800	-2.40389500	-2.06330800
C	-1.83861000	-1.15232900	-2.55685400
C	-2.40389500	1.13632800	-2.06330800
C	-1.15232900	1.83861000	-2.55685400
C	-0.65003400	3.39590600	-0.81502700
C	3.39590600	0.65003400	-0.81502700
C	0.65003400	-3.39590600	-0.81502700
C	-3.39590600	-0.65003400	-0.81502700
H	-0.48511600	4.25242000	-1.49046400
H	-1.72855000	3.31441700	-0.67052800
H	3.31441700	1.72855000	-0.67052800
H	4.25242000	0.48511600	-1.49046400
H	1.72855000	-3.31441700	-0.67052800
H	0.48511600	-4.25242000	-1.49046400
H	-4.25242000	-0.48511600	-1.49046400
H	-3.31441700	-1.72855000	-0.67052800
H	1.74395000	2.88328700	-1.29341200
H	1.07664400	3.11837100	-2.90212500
H	2.75183700	1.46012200	-3.09268900
H	1.21778300	0.65125600	-3.30014600
H	3.11837100	-1.07664400	-2.90212500
H	2.88328700	-1.74395000	-1.29341200
H	1.46012200	-2.75183700	-3.09268900
H	0.65125600	-1.21778300	-3.30014600
H	-1.07664400	-3.11837100	-2.90212500
H	-1.74395000	-2.88328700	-1.29341200
H	-1.21778300	-0.65125600	-3.30014600
H	-2.75183700	-1.46012200	-3.09268900
H	-2.88328700	1.74395000	-1.29341200
H	-3.11837100	1.07664400	-2.90212500
H	-0.65125600	1.21778300	-3.30014600
H	-1.46012200	2.75183700	-3.09268900
C	-3.05256300	-0.72848900	1.72584900
C	-0.72848900	3.05256300	1.72584900
C	3.05256300	0.72848900	1.72584900
C	0.72848900	-3.05256300	1.72584900
H	-1.01872400	-3.26889500	0.57171200
H	-0.07745600	-4.74849800	0.69344700
H	3.26889500	-1.01872400	0.57171200
H	4.74849800	-0.07745600	0.69344700
H	1.01872400	3.26889500	0.57171200
H	0.07745600	4.74849800	0.69344700
H	-3.26889500	1.01872400	0.57171200
H	-4.74849800	0.07745600	0.69344700

Pb(DOTAM)$^{2+}$ TSAP -1559.571192 Hartree

Pb	0.00000000	-0.00000000	0.51388693
N	1.45734091	-1.55027490	-1.11488935
N	1.55027490	1.45734091	-1.11488935
N	-1.45734091	1.55027490	-1.11488935
N	-1.55027490	-1.45734091	-1.11488935
O	2.38969990	-0.92980695	1.41092810
O	0.92980695	2.38969990	1.41092810
O	-2.38969990	0.92980695	1.41092810
O	-0.92980695	-2.38969990	1.41092810
N	3.51961801	-2.87138353	1.58492270
N	2.87138353	3.51961801	1.58492270
N	-3.51961801	2.87138353	1.58492270
N	-2.87138353	-3.51961801	1.58492270
C	2.55485629	-0.75783073	-1.69475317
H	3.01046097	-1.29491462	-2.53779982
H	3.33388674	-0.65117536	-0.93877093
C	2.11488963	0.61010198	-2.17675712
H	2.97497533	1.10694244	-2.64650822
H	1.36244682	0.50574669	-2.95967225
C	0.75783073	2.55485629	-1.69475317
H	1.29491462	3.01046097	-2.53779982
H	0.65117536	3.33388674	-0.93877093
C	-0.61010198	2.11488963	-2.17675712
H	-1.10694244	2.97497533	-2.64650822
H	-0.50574669	1.36244682	-2.95967225
C	-2.55485629	0.75783073	-1.69475317
H	-3.33388674	0.65117536	-0.93877093
H	-3.01046097	1.29491462	-2.53779982
C	-2.11488963	-0.61010198	-2.17675712
H	-1.36244682	-0.50574669	-2.95967225
H	-2.97497533	-1.10694244	-2.64650822
C	-0.75783073	-2.55485629	-1.69475317
H	-1.29491462	-3.01046097	-2.53779982
H	-0.65117536	-3.33388674	-0.93877093
C	0.61010198	-2.11488963	-2.17675712
H	1.10694244	-2.97497533	-2.64650822
H	0.50574669	-1.36244682	-2.95967225
C	1.99414962	-2.60893435	-0.26681846
H	2.69590174	-3.24937808	-0.81910601
H	1.17423378	-3.24417299	0.08095338
C	2.65823868	-2.05392545	0.98039442
H	3.94157032	-2.59028191	2.45657144
H	3.74208835	-3.78162812	1.21583464
C	2.60893435	1.99414962	-0.26681846
H	3.24937808	2.69590174	-0.81910601
H	3.24417299	1.17423378	0.08095338
C	2.05392545	2.65823868	0.98039442
H	2.59028191	3.94157032	2.45657144
H	3.78162812	3.74208835	1.21583464
C	-1.99414962	2.60893435	-0.26681846
H	-2.69590174	3.24937808	-0.81910601
H	-1.17423378	3.24417299	0.08095338
C	-2.65823868	2.05392545	0.98039442
H	-3.94157032	2.59028191	2.45657144
H	-3.74208835	3.78162812	1.21583464
C	-2.60893435	-1.99414962	-0.26681846
H	-3.24937808	-2.69590174	-0.81910601
H	-3.24417299	-1.17423378	0.08095338
C	-2.05392545	-2.65823868	0.98039442
H	-2.59028191	-3.94157032	2.45657144
H	-3.78162812	-3.74208835	1.21583464

Pb(DOTAM)$^{2+}$ SAP -1559.56632 Hartree

Pb	0.00000000	0.00000000	0.67646118
N	-1.28426424	1.72537403	-1.00552572
N	1.72537403	1.28426424	-1.00552572
N	1.28426424	-1.72537403	-1.00552572
N	-1.72537403	-1.28426424	-1.00552572
O	-2.53105821	0.67925457	1.35315322
O	0.67925457	2.53105821	1.35315322
O	2.53105821	-0.67925457	1.35315322
O	-0.67925457	-2.53105821	1.35315322
C	-3.01904333	1.66274791	0.79360028
C	1.66274791	3.01904333	0.79360028
C	3.01904333	-1.66274791	0.79360028
C	-1.66274791	-3.01904333	0.79360028
C	-0.30481327	2.65162633	-1.60308366
C	0.97324116	1.97635264	-2.06041470
C	2.65162633	0.30481327	-1.60308366
C	1.97635264	-0.97324116	-2.06041470
C	0.30481327	-2.65162633	-1.60308366
C	-0.97324116	-1.97635264	-2.06041470
C	-2.65162633	-0.30481327	-1.60308366
C	-1.97635264	0.97324116	-2.06041470
C	-2.22581951	2.49488946	-0.19604167
C	2.49488946	2.22581951	-0.19604167
C	2.22581951	-2.49488946	-0.19604167
C	-2.49488946	-2.22581951	-0.19604167
H	-1.64857535	3.18792213	0.42450137
H	-2.89616749	3.09380691	-0.82814464
H	3.09380691	2.89616749	-0.82814464
H	3.18792213	1.64857535	0.42450137
H	2.89616749	-3.09380691	-0.82814464
H	1.64857535	-3.18792213	0.42450137
H	-3.18792213	-1.64857535	0.42450137
H	-3.09380691	-2.89616749	-0.82814464
H	-0.08330131	3.42847088	-0.87218061
H	-0.74272513	3.16429202	-2.47105963
H	1.60629662	2.73812051	-2.53715465
H	0.74541777	1.24678530	-2.83852875
H	3.16429202	0.74272513	-2.47105963
H	3.42847088	0.08330131	-0.87218061
H	2.73812051	-1.60629662	-2.53715465
H	1.24678530	-0.74541777	-2.83852875
H	0.74272513	-3.16429202	-2.47105963
H	0.08330131	-3.42847088	-0.87218061
H	-0.74541777	-1.24678530	-2.83852875
H	-1.60629662	-2.73812051	-2.53715465
H	-3.42847088	-0.08330131	-0.87218061
H	-3.16429202	-0.74272513	-2.47105963
H	-1.24678530	0.74541777	-2.83852875
H	-2.73812051	1.60629662	-2.53715465
N	-4.24974774	2.09932404	1.05855630
H	-4.78990685	1.64725605	1.78144827
H	-4.64495807	2.89702528	0.58709935
N	2.09932404	4.24974774	1.05855630
H	1.64725605	4.78990685	1.78144827
H	2.89702528	4.64495807	0.58709935
N	4.24974774	-2.09932404	1.05855630
H	4.78990685	-1.64725605	1.78144827
H	4.64495807	-2.89702528	0.58709935
N	-2.09932404	-4.24974774	1.05855630
H	-1.64725605	-4.78990685	1.78144827
H	-2.89702528	-4.64495807	0.58709935

Pb(DO2A2AM) TSAP -1598.37616 Hartree

Pb	0.00000000	0.00000000	0.54042100
N	-0.97283600	1.90309100	-1.11710300
N	-1.88683500	-0.94212700	-1.11504300
N	0.97283600	-1.90309100	-1.11710300
N	1.88683500	0.94212700	-1.11504300
O	-2.02408000	1.63811000	1.43785800
O	-1.46665200	-2.04382000	1.37630300
O	2.02408000	-1.63811000	1.43785800
O	1.46665200	2.04382000	1.37630300
N	-2.52477900	3.83183600	1.55713900
N	2.52477900	-3.83183600	1.55713900
C	-2.25584100	1.45921300	-1.67987500
H	-2.55594500	2.10816000	-2.51549400
H	-3.01952000	1.56906800	-0.90850200
C	-2.22794000	0.02191300	-2.16509800
H	-3.20930600	-0.21462800	-2.60250200
H	-1.50411800	-0.08485400	-2.97538200
C	-1.43626100	-2.21145800	-1.69945300
H	-2.08189800	-2.49753800	-2.54279200
H	-1.55249100	-2.98627900	-0.94115900
C	0.00000000	-2.17614600	-2.18279900
H	0.22933400	-3.13302700	-2.67471400
H	0.11805600	-1.40726800	-2.94778600
C	2.25584100	-1.45921300	-1.67987500
H	3.01952000	-1.56906800	-0.90850200
H	2.55594500	-2.10816000	-2.51549400
C	2.22794000	-0.02191300	-2.16509800
H	1.50411800	0.08485400	-2.97538200
H	3.20930600	0.21462800	-2.60250200
C	1.43626100	2.21145800	-1.69945300
H	2.08189800	2.49753800	-2.54279200
H	1.55249100	2.98627900	-0.94115900
C	0.00000000	2.17614600	-2.18279900
H	-0.22933400	3.13302700	-2.67471400
H	-0.11805600	1.40726800	-2.94778600
C	-1.15809200	3.08173100	-0.28320100
H	-1.63173700	3.90289600	-0.84111000
H	-0.17994300	3.42999200	0.06212900
C	-1.94880300	2.77626900	0.97466600
H	-2.99810400	3.71405200	2.43936400
H	-2.46465900	4.75696500	1.16450500
C	-3.02625700	-1.18062000	-0.22801800
H	-3.83629100	-1.69996200	-0.75922500
H	-3.42305400	-0.21830700	0.10704400
C	-2.68428300	-1.97056000	1.04629700
C	1.15809200	-3.08173100	-0.28320100
H	1.63173700	-3.90289600	-0.84111000
H	0.17994300	-3.42999200	0.06212900
C	1.94880300	-2.77626900	0.97466600
H	2.99810400	-3.71405200	2.43936400
H	2.46465900	-4.75696500	1.16450500
C	3.02625700	1.18062000	-0.22801800
H	3.83629100	1.69996200	-0.75922500
H	3.42305400	0.21830700	0.10704400
C	2.68428300	1.97056000	1.04629700
O	-3.65064700	-2.44988200	1.66187900
O	3.65064700	2.44988200	1.66187900

Pb(DO2A2AM) SAP -1598.36895 Hartree

Pb	-0.00000000	0.00000000	0.67506386
N	0.88485222	1.93801827	-1.00433389
N	1.97770406	-0.87826105	-1.01355152
N	-0.88485222	-1.93801827	-1.00433389
N	-1.97770406	0.87826105	-1.01355152
O	-0.66269727	2.45062548	1.34545876
O	2.57124553	0.59601282	1.39228045
O	0.66269727	-2.45062548	1.34545876
O	-2.57124553	-0.59601282	1.39228045
O	0.16446689	-4.62580593	1.19318489
O	-0.16446689	4.62580593	1.19318489
C	-0.03758165	3.43491568	0.86092565
C	3.46111781	-0.01904131	0.80437165
C	0.03758165	-3.43491568	0.86092565
C	-3.46111781	0.01904131	0.80437165
C	2.18073121	1.55897342	-1.58627738
C	2.24048657	0.11826358	-2.05956759
C	1.54294102	-2.14945037	-1.61587108
C	0.09903896	-2.13938623	-2.07578160
C	-2.18073121	-1.55897342	-1.58627738
C	-2.24048657	-0.11826358	-2.05956759
C	-1.54294102	2.14945037	-1.61587108
C	-0.09903896	2.13938623	-2.07578160
C	1.04510298	3.14863788	-0.19247970
C	3.15334356	-1.12461729	-0.18639726
C	-1.04510298	-3.14863788	-0.19247970
C	-3.15334356	1.12461729	-0.18639726
H	1.97071834	3.04155902	0.38145539
H	1.15611867	4.03450734	-0.83228815
H	4.03605257	-1.34952151	-0.80224694
H	2.94097685	-1.99956698	0.43665339
H	-1.15611867	-4.03450734	-0.83228815
H	-1.97071834	-3.04155902	0.38145539
H	-2.94097685	1.99956698	0.43665339
H	-4.03605257	1.34952151	-0.80224694
H	2.95042237	1.74096637	-0.83693139
H	2.42599448	2.20636626	-2.44177475
H	3.22860698	-0.05584074	-2.51112681
H	1.51393034	-0.03609520	-2.85878619
H	2.17581255	-2.40054976	-2.47972711
H	1.69081271	-2.93820390	-0.87968937
H	-0.09853013	-3.09229169	-2.59053719
H	-0.04394305	-1.35696048	-2.82363068
H	-2.42599448	-2.20636626	-2.44177475
H	-2.95042237	-1.74096637	-0.83693139
H	-1.51393034	0.03609520	-2.85878619
H	-3.22860698	0.05584074	-2.51112681
H	-1.69081271	2.93820390	-0.87968937
H	-2.17581255	2.40054976	-2.47972711
H	0.04394305	1.35696048	-2.82363068
H	0.09853013	3.09229169	-2.59053719
N	4.75873712	0.19783198	1.03917519
H	5.03059211	0.84942011	1.75911882
H	5.48037091	-0.30075589	0.54540964
N	-4.75873712	-0.19783198	1.03917519
H	-5.03059211	-0.84942011	1.75911882
H	-5.48037091	0.30075589	0.54540964

Pb(DO2A2Py) TSAP -1754.959217 Hartree

Pb	0.00000000	-0.00000000	0.24709299
N	-1.90657999	0.98370248	-1.42787425
N	1.00341700	1.86806850	-1.42326560
N	1.90657999	-0.98370248	-1.42787425
N	-1.00341700	-1.86806850	-1.42326560
N	-2.62912390	0.16762826	1.17699902
N	2.62912390	-0.16762826	1.17699902
O	0.15618308	2.49041927	1.13232524
O	-0.15618308	-2.49041927	1.13232524
O	1.74619708	4.04762035	1.37724272
O	-1.74619708	-4.04762035	1.37724272
C	-1.26531403	1.79333417	-2.46949951
H	-2.00507805	2.45432703	-2.94669268
H	-0.91500605	1.12001994	-3.25339732
C	-0.11566502	2.64522133	-1.96773680
H	0.22858795	3.28598109	-2.79390400
H	-0.46683400	3.31182157	-1.17892299
C	1.79998097	1.26623819	-2.49558245
H	2.44496296	2.02159605	-2.96992869
H	1.11927895	0.91950195	-3.27533732
C	2.67328400	0.11730233	-2.02486513
H	3.27197297	-0.24266692	-2.87497804
H	3.38324402	0.47840456	-1.27903926
C	1.26531403	-1.79333417	-2.46949951
H	2.00507805	-2.45432703	-2.94669268
H	0.91500605	-1.12001994	-3.25339732
C	0.11566502	-2.64522133	-1.96773680
H	-0.22858795	-3.28598109	-2.79390400
H	0.46683400	-3.31182157	-1.17892299
C	-1.79998097	-1.26623819	-2.49558245
H	-1.11927895	-0.91950195	-3.27533732
H	-2.44496296	-2.02159605	-2.96992869
C	-2.67328400	-0.11730233	-2.02486513
H	-3.27197297	0.24266692	-2.87497804
H	-3.38324402	-0.47840456	-1.27903926
C	-2.76366297	1.82598073	-0.59088447
H	-3.56023699	2.29211154	-1.18975959
H	-2.14666396	2.63192886	-0.18031673
C	-3.37973893	1.08927907	0.56882177
C	-4.66771492	1.38876420	1.00224372
H	-5.25850394	2.13068103	0.47727951
C	-5.17691688	0.71896252	2.10598294
H	-6.17817587	0.93449063	2.46246590
C	-4.39174385	-0.23630628	2.73847620
H	-4.75236882	-0.78717202	3.59861038
C	-3.12302287	-0.47853342	2.23304423
H	-2.47240485	-1.22276328	2.68430944
C	1.82737602	2.72740276	-0.57519089
H	2.12364800	3.64117560	-1.11076017
H	2.74909404	2.19914685	-0.31836376
C	1.17858506	3.13054916	0.75925101
C	2.76366297	-1.82598073	-0.59088447
H	3.56023699	-2.29211154	-1.18975959
H	2.14666396	-2.63192886	-0.18031673
C	3.37973893	-1.08927907	0.56882177
C	4.66771492	-1.38876420	1.00224372
H	5.25850394	-2.13068103	0.47727951
C	5.17691688	-0.71896252	2.10598294
H	6.17817587	-0.93449063	2.46246590
C	4.39174385	0.23630628	2.73847620
H	4.75236882	0.78717202	3.59861038
C	3.12302287	0.47853342	2.23304423
H	2.47240485	1.22276328	2.68430944
C	-1.82737602	-2.72740276	-0.57519089
H	-2.12364800	-3.64117560	-1.11076017
H	-2.74909404	-2.19914685	-0.31836376
C	-1.17858506	-3.13054916	0.75925101

Pb(DO2A2Py) SAP -1754.952457 Hartree

Pb	0.00000000	-0.00000000	0.47038493
N	-1.53024518	1.53703010	-1.23862780
N	1.55458612	1.48733328	-1.26611472
N	1.53024518	-1.53703010	-1.23862780
N	-1.55458612	-1.48733328	-1.26611472
N	2.68417587	-0.45367704	1.23620347
N	-2.68417587	0.45367704	1.23620347
O	0.40509777	2.50656430	1.14697260
O	-0.40509777	-2.50656430	1.14697260
O	-1.61151257	-4.37725129	0.94632942
O	1.61151257	4.37725129	0.94632942
C	-3.33935121	1.33754505	0.47730658
C	1.29470591	3.22281311	0.61022428
C	3.33935121	-1.33754505	0.47730658
C	-1.29470591	-3.22281311	0.61022428
C	-0.64546417	2.53601660	-1.85733339
C	0.67215966	1.96179775	-2.33730718
C	2.62733255	0.65719329	-1.82998016
C	2.18104797	-0.72566829	-2.27601046
C	0.64546417	-2.53601660	-1.85733339
C	-0.67215966	-1.96179775	-2.33730718
C	-2.62733255	-0.65719329	-1.82998016
C	-2.18104797	0.72566829	-2.27601046
C	-2.50108174	2.23762272	-0.38843677
C	2.13112044	2.61356396	-0.52739505
C	2.50108174	-2.23762272	-0.38843677
C	-2.13112044	-2.61356396	-0.52739505
H	-1.92327746	2.88727543	0.27633982
H	-3.16038960	2.87209256	-0.99868946
H	2.42493744	3.42070188	-1.21239706
H	3.04851338	2.26729829	-0.04179988
H	3.16038960	-2.87209256	-0.99868946
H	1.92327746	-2.88727543	0.27633982
H	-3.04851338	-2.26729829	-0.04179988
H	-2.42493744	-3.42070188	-1.21239706
H	-0.46194085	3.32314789	-1.12596003
H	-1.14696027	3.01325842	-2.71242034
H	1.18712414	2.73375517	-2.93045509
H	0.48121708	1.13052341	-3.01909913
H	3.08786363	1.15412318	-2.69850413
H	3.40962073	0.56780084	-1.07752867
H	3.06132101	-1.25353587	-2.67345209
H	1.48798219	-0.63304587	-3.11398081
H	1.14696027	-3.01325842	-2.71242034
H	0.46194085	-3.32314789	-1.12596003
H	-0.48121708	-1.13052341	-3.01909913
H	-1.18712414	-2.73375517	-2.93045509
H	-3.40962073	-0.56780084	-1.07752867
H	-3.08786363	-1.15412318	-2.69850413
H	-1.48798219	0.63304587	-3.11398081
H	-3.06132101	1.25353587	-2.67345209
C	4.72532935	-1.44909085	0.51053604
H	5.22673023	-2.17093508	-0.12366798
C	-4.72532935	1.44909085	0.51053604
H	-5.22673023	2.17093508	-0.12366798
C	3.37894409	0.34859229	2.04044564
H	2.80017564	1.05750847	2.62628011
C	-3.37894409	-0.34859229	2.04044564
H	-2.80017564	-1.05750847	2.62628011
C	5.44663193	-0.61418674	1.35427128
H	6.52843030	-0.67775099	1.39375520
C	4.76362715	0.30679541	2.13654122
H	5.28568875	0.98179793	2.80369544
C	-4.76362715	-0.30679541	2.13654122
H	-5.28568875	-0.98179793	2.80369544
C	-5.44663193	0.61418674	1.35427128
H	-6.52843030	0.67775099	1.39375520

Pb(DO3APA)$^{2-}$ TSAP -1884.051662 Hartree

Pb	-0.23940500	-0.05472500	-0.43780200
N	-2.57165700	-1.06647800	0.73731300
N	0.13170800	-1.42559000	2.04269800
N	0.71262300	1.53141900	1.80585600
N	-2.02881300	1.88538500	0.45730000
N	2.43657800	0.78988300	-0.39354200
O	0.36634600	-2.74394400	-0.36699000
O	-0.38782900	2.24249100	-1.75712000
O	-2.19672700	-0.78283000	-2.00966700
O	1.56601300	-0.64386200	-2.51777400
O	2.11068300	-3.94857500	0.35815500
O	-1.83377700	3.69182400	-2.65966900
O	-3.58818300	-2.48437700	-2.41733700
O	3.61733700	-1.50017600	-2.82369700
C	-2.35743000	-1.56925700	2.09346500
H	-3.16729200	-2.25976600	2.38219800
H	-2.42347200	-0.72655900	2.78441200
C	-1.03138300	-2.28195100	2.28144600
H	-1.00267000	-2.70171300	3.30097200
H	-0.97067600	-3.12250000	1.59038000
C	0.35179500	-0.52845900	3.17354600
H	0.79886000	-1.07261600	4.02270300
H	-0.62038100	-0.17600600	3.52379100
C	1.24045500	0.65885800	2.85586900
H	1.41162900	1.22571300	3.78606800
H	2.22187900	0.30360500	2.53275200
C	-0.39599400	2.34712600	2.30222100
H	-0.01451600	3.19252400	2.89845100
H	-0.98397000	1.73967000	2.99181700
C	-1.29558500	2.90347700	1.21314100
H	-1.99610700	3.61798100	1.67629600
H	-0.69671100	3.47182500	0.49862100
C	-3.11315300	1.30807000	1.25222800
H	-2.76381500	1.18944000	2.27921900
H	-3.96843200	2.00154300	1.30101500
C	-3.60128600	-0.02784600	0.72217500
H	-4.48115400	-0.33915800	1.30901500
H	-3.93969800	0.08910700	-0.30893700
C	-2.92720800	-2.14726200	-0.17306300
H	-3.91399900	-2.56985900	0.07084000
H	-2.18805400	-2.94696100	-0.06831600
C	-2.91213700	-1.76450400	-1.65997900
C	1.31077300	-2.23971900	1.77752800
H	1.51573600	-2.93283200	2.60935100
H	2.18618800	-1.58811000	1.69214000
C	1.24951400	-3.05244100	0.47257400
C	1.77968100	2.38633400	1.28912500
H	2.23184800	2.98461500	2.09649700
H	1.34144300	3.08472000	0.57058600
C	2.85125000	1.60578600	0.57534600
C	4.19967400	1.75266800	0.88421400
H	4.50734200	2.42171800	1.67981600
C	5.13122900	1.01974400	0.15881900
H	6.18928900	1.10695800	0.38206600
C	4.69061700	0.17801900	-0.85125300
H	5.37322600	-0.41168000	-1.44927800
C	3.32337000	0.09626000	-1.10603100
C	-2.55634500	2.47192400	-0.77010500
H	-3.16667600	3.36198000	-0.55355000
H	-3.20887900	1.74154400	-1.25699600
C	-1.49476500	2.84298900	-1.81329400
C	2.78464400	-0.76722700	-2.25075800

Pb(DO3APA)$^{2-}$ SAP -1884.044583 Hartree

Pb	-0.25618300	-0.06763400	-0.52401800
N	0.44863300	1.95438500	1.43370800
N	-2.41797700	1.42283900	0.42662700
N	-2.09777900	-1.55899300	0.95637700
N	0.77012400	-1.04571900	1.93012200
N	2.36012800	0.93275700	-0.54641800
O	-0.63263000	2.31386000	-1.63770500
O	0.50767900	-2.65546200	-0.41001800
O	-2.34910800	-0.91219800	-1.83729600
O	1.61528500	-0.57880800	-2.65194100
O	1.89625500	-4.17267800	0.46964700
O	-1.98138200	4.05961500	-2.00082000
O	-4.06361500	-2.33760400	-1.96902300
O	3.61658900	-1.59678600	-2.67730400
C	2.71989900	1.79524600	0.40282700
C	-1.70728900	2.95483100	-1.49125000
C	-3.08835600	-1.83025200	-1.38372800
C	1.32125300	-3.06473000	0.45542000
C	-0.68486600	2.88127200	1.52224700
C	-2.03767500	2.19973000	1.60754400
C	-3.51202500	0.50296400	0.75839800
C	-3.08437300	-0.68784300	1.59524800
C	-1.38874100	-2.34883400	1.96903000
C	-0.34000600	-1.56319800	2.73472500
C	1.46440500	0.01384200	2.66869300
C	0.71117600	1.33167000	2.73192500
C	1.62117100	2.67617600	0.93687700
C	-2.82747500	2.29452100	-0.67279700
C	-2.72163400	-2.44467700	-0.02366700
C	1.71785400	-2.10443500	1.58877800
H	1.28454200	3.29414700	0.09979100
H	2.02695200	3.34759600	1.71008300
H	-3.51550800	3.07655000	-0.31973400
H	-3.37277600	1.67904900	-1.39541900
H	-3.61704000	-2.92824600	0.39382400
H	-1.99607600	-3.22882000	-0.26129600
H	2.64374900	-1.63055000	1.24454200
H	1.97785700	-2.70174800	2.47569600
H	-0.65557400	3.52776500	0.64495600
H	-0.58290000	3.53933500	2.40105200
H	-2.79435200	2.97737600	1.80515200
H	-2.05583400	1.53612400	2.47374000
H	-4.31375500	1.03087000	1.30091100
H	-3.94472700	0.15357900	-0.17890400
H	-3.98863400	-1.26377500	1.85291700
H	-2.67122100	-0.34080100	2.54409200
H	-2.09772700	-2.76565100	2.70316900
H	-0.92268900	-3.19458200	1.46659900
H	-0.81735500	-0.71912700	3.23607800
H	0.05077900	-2.21100200	3.53748000
H	2.43457100	0.16911900	2.19668700
H	1.67235400	-0.30408500	3.70422200
H	-0.24429300	1.18136800	3.23723800
H	1.28598700	2.01812800	3.37556700
C	4.04158500	1.91479800	0.82148400
H	4.30835900	2.62973200	1.59163200
C	3.27184900	0.15028800	-1.12124500
C	4.61162000	0.18909500	-0.74081900
H	5.31738000	-0.47354400	-1.22486800
C	4.99783100	1.08871900	0.24251400
H	6.03433700	1.14914500	0.55718200
C	2.78814900	-0.76558200	-2.25056700

Pb(DO4Py)$^{2+}$ TSAP -1872.74181 Hartree

Pb	0.00000000	-0.00000000	-0.07769706
N	1.05023210	-1.86900033	-1.70667206
N	-1.86900033	-1.05023210	-1.70667206
N	-1.05023210	1.86900033	-1.70667206
N	1.86900033	1.05023210	-1.70667206
N	2.05675786	-1.57130483	0.89541894
N	-1.57130483	-2.05675786	0.89541894
N	-2.05675786	1.57130483	0.89541894
N	1.57130483	2.05675786	0.89541894
C	0.08579758	-2.19829133	-2.76464206
H	0.35596850	-3.15077442	-3.24362806
H	0.16638793	-1.43894966	-3.54371806
C	-1.34918422	-2.29962302	-2.28160706
H	-1.97701981	-2.62401310	-3.12417806
H	-1.42974417	-3.07748760	-1.52062906
C	-2.19829133	-0.08579758	-2.76464206
H	-3.15077442	-0.35596850	-3.24362806
H	-1.43894966	-0.16638793	-3.54371806
C	-2.29962302	1.34918422	-2.28160706
H	-2.62401310	1.97701981	-3.12417806
H	-3.07748760	1.42974417	-1.52062906
C	-0.08579758	2.19829133	-2.76464206
H	-0.35596850	3.15077442	-3.24362806
H	-0.16638793	1.43894966	-3.54371806
C	1.34918422	2.29962302	-2.28160706
H	1.97701981	2.62401310	-3.12417806
H	1.42974417	3.07748760	-1.52062906
C	2.19829133	0.08579758	-2.76464206
H	1.43894966	0.16638793	-3.54371806
H	3.15077442	0.35596850	-3.24362806
C	2.29962302	-1.34918422	-2.28160706
H	2.62401310	-1.97701981	-3.12417806
H	3.07748760	-1.42974417	-1.52062906
C	1.32443902	-3.05497165	-0.89286706
H	1.81904233	-3.83187940	-1.49430306
H	0.36867407	-3.47372460	-0.56287906
C	2.15126044	-2.77987572	0.33644494
C	2.94821231	-3.78092855	0.88432994
H	3.01559798	-4.74805330	0.39929094
C	3.64849805	-3.51651459	2.05183394
H	4.27411432	-4.28107050	2.49880994
C	3.54486155	-2.25881554	2.63375794
H	4.07979329	-2.00870418	3.54168394
C	2.73776352	-1.31777806	2.01527594
H	2.62806541	-0.31777446	2.42603594
C	-3.05497165	-1.32443902	-0.89286706
H	-3.83187940	-1.81904233	-1.49430306
H	-3.47372460	-0.36867407	-0.56287906
C	-2.77987572	-2.15126044	0.33644494
C	-3.78092855	-2.94821231	0.88432994
H	-4.74805330	-3.01559798	0.39929094
C	-3.51651459	-3.64849805	2.05183394
H	-4.28107050	-4.27411432	2.49880994
C	-2.25881554	-3.54486155	2.63375794
H	-2.00870418	-4.07979329	3.54168394
C	-1.31777806	-2.73776352	2.01527594
H	-0.31777446	-2.62806541	2.42603594
C	-1.32443902	3.05497165	-0.89286706
H	-1.81904233	3.83187940	-1.49430306
H	-0.36867407	3.47372460	-0.56287906
C	-2.15126044	2.77987572	0.33644494
C	-2.94821231	3.78092855	0.88432994
H	-3.01559798	4.74805330	0.39929094
C	-3.64849805	3.51651459	2.05183394
H	-4.27411432	4.28107050	2.49880994
C	-3.54486155	2.25881554	2.63375794
H	-4.07979329	2.00870418	3.54168394
C	-2.73776352	1.31777806	2.01527594
H	-2.62806541	0.31777446	2.42603594
C	3.05497165	1.32443902	-0.89286706
H	3.83187940	1.81904233	-1.49430306
H	3.47372460	0.36867407	-0.56287906
C	2.77987572	2.15126044	0.33644494
C	3.78092855	2.94821231	0.88432994
H	4.74805330	3.01559798	0.39929094
C	3.51651459	3.64849805	2.05183394
H	4.28107050	4.27411432	2.49880994
C	2.25881554	3.54486155	2.63375794
H	2.00870418	4.07979329	3.54168394
C	1.31777806	2.73776352	2.01527594
H	0.31777446	2.62806541	2.42603594

Pb(DO4Py)$^{2+}$ SAP -1872.734574 Hartree

Pb	-0.00000000	0.00000000	-0.18856987
N	-2.17404072	-0.14399881	1.46574805
N	0.14399881	-2.17404072	1.46574805
N	2.17404072	0.14399881	1.46574805
N	-0.14399881	2.17404072	1.46574805
N	-1.31826691	-2.26877541	-1.03407983
N	2.26877541	-1.31826691	-1.03407983
N	1.31826691	2.26877541	-1.03407983
N	-2.26877541	1.31826691	-1.03407983
C	-3.36685602	1.20848404	-0.28054706
C	-1.20848404	-3.36685602	-0.28054706
C	3.36685602	-1.20848404	-0.28054706
C	1.20848404	3.36685602	-0.28054706
C	-2.21964041	-1.48087586	2.08124710
C	-0.87161554	-1.98986948	2.55322918
C	1.48087586	-2.21964041	2.08124710
C	1.98986948	-0.87161554	2.55322918
C	2.21964041	1.48087586	2.08124710
C	0.87161554	1.98986948	2.55322918
C	-1.48087586	2.21964041	2.08124710
C	-1.98986948	0.87161554	2.55322918
C	-3.39950603	0.05036802	0.67911599
C	-0.05036802	-3.39950603	0.67911599
C	3.39950603	-0.05036802	0.67911599
C	0.05036802	3.39950603	0.67911599
H	-3.55415843	-0.85466628	0.08361002
H	-4.27034623	0.14921352	1.34228095
H	-0.14921352	-4.27034623	1.34228095
H	0.85466628	-3.55415843	0.08361002
H	4.27034623	-0.14921352	1.34228095
H	3.55415843	0.85466628	0.08361002
H	-0.85466628	3.55415843	0.08361002
H	0.14921352	4.27034623	1.34228095
H	-2.64309517	-2.17323463	1.35374411
H	-2.90279544	-1.48322702	2.94324407
H	-1.03423288	-2.94793675	3.06863321
H	-0.46164776	-1.31133274	3.30335017
H	1.48322702	-2.90279544	2.94324407
H	2.17323463	-2.64309517	1.35374411
H	2.94793675	-1.03423288	3.06863321
H	1.31133274	-0.46164776	3.30335017
H	2.90279544	1.48322702	2.94324407
H	2.64309517	2.17323463	1.35374411
H	0.46164776	1.31133274	3.30335017
H	1.03423288	2.94793675	3.06863321
H	-2.17323463	2.64309517	1.35374411
H	-1.48322702	2.90279544	2.94324407
H	-1.31133274	0.46164776	3.30335017
H	-2.94793675	1.03423288	3.06863321
C	-2.10586017	-4.42179476	-0.38323877
H	-2.01369169	-5.27993457	0.27200027
C	4.42179476	-2.10586017	-0.38323877
H	5.27993457	-2.01369169	0.27200027
C	2.10586017	4.42179476	-0.38323877
H	2.01369169	5.27993457	0.27200027
C	-4.42179476	2.10586017	-0.38323877
H	-5.27993457	2.01369169	0.27200027
C	-2.27498497	-2.21441612	-1.96148287
H	-2.31230353	-1.30765021	-2.55862991
C	2.21441612	-2.27498497	-1.96148287
H	1.30765021	-2.31230353	-2.55862991
C	2.27498497	2.21441612	-1.96148287
H	2.31230353	1.30765021	-2.55862991
C	-2.21441612	2.27498497	-1.96148287
H	-1.30765021	2.31230353	-2.55862991
C	3.12900249	4.37142435	-1.31266493
H	3.82979906	5.19322657	-1.40894593
C	3.24416349	3.24575752	-2.11712787
H	4.02853868	3.15712886	-2.85866684
C	4.37142435	-3.12900249	-1.31266493
H	5.19322657	-3.82979906	-1.40894593
C	3.24575752	-3.24416349	-2.11712787
H	3.15712886	-4.02853868	-2.85866684
C	-3.24416349	-3.24575752	-2.11712787
H	-4.02853868	-3.15712886	-2.85866684
C	-3.12900249	-4.37142435	-1.31266493
H	-3.82979906	-5.19322657	-1.40894593
C	-3.24575752	3.24416349	-2.11712787
H	-3.15712886	4.02853868	-2.85866684
C	-4.37142435	3.12900249	-1.31266493
H	-5.19322657	3.82979906	-1.40894593

Pb(DO4Pyd)$^{2+}$ TSAP -1936.730059 Hartree

Pb	0.00000000	0.00000000	-0.10114401
N	1.36961133	1.64261108	-1.71940601
N	1.64261108	-1.36961133	-1.71940601
N	-1.36961133	-1.64261108	-1.71940601
N	-1.64261108	1.36961133	-1.71940601
N	0.65816456	2.51727614	0.79697699
N	2.51727614	-0.65816456	0.79697699
N	-0.65816456	-2.51727614	0.79697699
N	-2.51727614	0.65816456	0.79697699
N	-0.13288289	-2.97472687	1.92160799
N	-2.97472687	0.13288289	1.92160799
N	0.13288289	2.97472687	1.92160799
N	2.97472687	-0.13288289	1.92160799
C	2.02516728	0.86286102	-2.77887401
H	2.81968545	1.45644170	-3.25345601
H	1.28846145	0.66979999	-3.55958701
C	2.62861358	-0.44314383	-2.29865101
H	3.14589518	-0.91812875	-3.14416801
H	3.38973878	-0.24420800	-1.54327101
C	0.86286102	-2.02516728	-2.77887401
H	1.45644170	-2.81968545	-3.25345601
H	0.66979999	-1.28846145	-3.55958701
C	-0.44314383	-2.62861358	-2.29865101
H	-0.91812875	-3.14589518	-3.14416801
H	-0.24420800	-3.38973878	-1.54327101
C	-2.02516728	-0.86286102	-2.77887401
H	-2.81968545	-1.45644170	-3.25345601
H	-1.28846145	-0.66979999	-3.55958701
C	-2.62861358	0.44314383	-2.29865101
H	-3.14589518	0.91812875	-3.14416801
H	-3.38973878	0.24420800	-1.54327101
C	-0.86286102	2.02516728	-2.77887401
H	-0.66979999	1.28846145	-3.55958701
H	-1.45644170	2.81968545	-3.25345601
C	0.44314383	2.62861358	-2.29865101
H	0.91812875	3.14589518	-3.14416801
H	0.24420800	3.38973878	-1.54327101
C	2.37658534	2.31968024	-0.90207501
H	2.89787965	3.09290781	-1.48598801
H	3.13280584	1.58862967	-0.60165901
C	1.83687486	2.93902153	0.35769999
C	2.57619304	3.90138493	1.05267099
H	3.53169169	4.24443257	0.67254799
C	2.04152395	4.38284426	2.22246499
H	2.55112998	5.12768357	2.82139099
C	0.80056883	3.87914430	2.61931899
H	0.31796404	4.21389978	3.53090899
C	2.31968024	-2.37658534	-0.90207501
H	3.09290781	-2.89787965	-1.48598801
H	1.58862967	-3.13280584	-0.60165901
C	2.93902153	-1.83687486	0.35769999
C	3.90138493	-2.57619304	1.05267099
H	4.24443257	-3.53169169	0.67254799
C	4.38284426	-2.04152395	2.22246499
H	5.12768357	-2.55112998	2.82139099
C	3.87914430	-0.80056883	2.61931899
H	4.21389978	-0.31796404	3.53090899
C	-2.37658534	-2.31968024	-0.90207501
H	-2.89787965	-3.09290781	-1.48598801
H	-3.13280584	-1.58862967	-0.60165901
C	-1.83687486	-2.93902153	0.35769999
C	-2.57619304	-3.90138493	1.05267099
H	-3.53169169	-4.24443257	0.67254799
C	-2.04152395	-4.38284426	2.22246499
H	-2.55112998	-5.12768357	2.82139099
C	-0.80056883	-3.87914430	2.61931899
H	-0.31796404	-4.21389978	3.53090899
C	-2.31968024	2.37658534	-0.90207501
H	-3.09290781	2.89787965	-1.48598801
H	-1.58862967	3.13280584	-0.60165901
C	-2.93902153	1.83687486	0.35769999
C	-3.90138493	2.57619304	1.05267099
H	-4.24443257	3.53169169	0.67254799
C	-4.38284426	2.04152395	2.22246499
H	-5.12768357	2.55112998	2.82139099
C	-3.87914430	0.80056883	2.61931899
H	-4.21389978	0.31796404	3.53090899

Pb(DO4Pyd)$^{2+}$ SAP -1936.720484 Hartree

Pb	0.00000000	0.00000000	0.24346400
N	0.00000000	2.17516900	-1.47449400
N	2.17516900	0.00000000	-1.47449400
N	0.00000000	-2.17516900	-1.47449400
N	-2.17516900	0.00000000	-1.47449400
N	2.18155000	1.57534500	0.93214000
N	1.57534500	-2.18155000	0.93214000
N	-2.18155000	-1.57534500	0.93214000
N	-1.57534500	2.18155000	0.93214000
N	-2.51642200	2.03479900	1.84636400
N	-2.03479900	-2.51642200	1.84636400
N	2.51642200	-2.03479900	1.84636400
N	2.03479900	2.51642200	1.84636400
C	-1.43220700	3.31490700	0.25461400
C	3.31490700	1.43220700	0.25461400
C	1.43220700	-3.31490700	0.25461400
C	-3.31490700	-1.43220700	0.25461400
C	1.33738200	2.30885200	-2.07944100
C	1.95006500	0.99715200	-2.53287100
C	2.30885200	-1.33738200	-2.07944100
C	0.99715200	-1.95006500	-2.53287100
C	-1.33738200	-2.30885200	-2.07944100
C	-1.95006500	-0.99715200	-2.53287100
C	-2.30885200	1.33738200	-2.07944100
C	-0.99715200	1.95006500	-2.53287100
C	-0.28724500	3.39825500	-0.71664700
C	3.39825500	0.28724500	-0.71664700
C	0.28724500	-3.39825500	-0.71664700
C	-3.39825500	-0.28724500	-0.71664700
H	0.60009200	3.64134500	-0.12441300
H	-0.45710600	4.24448200	-1.39760400
H	4.24448200	0.45710600	-1.39760400
H	3.64134500	-0.60009200	-0.12441300
H	0.45710600	-4.24448200	-1.39760400
H	-0.60009200	-3.64134500	-0.12441300
H	-3.64134500	0.60009200	-0.12441300
H	-4.24448200	-0.45710600	-1.39760400
H	1.98894600	2.79728000	-1.35630600
H	1.29558000	2.97636000	-2.95260100
H	2.89962300	1.22681200	-3.03781800
H	1.31192900	0.54046900	-3.29057700
H	2.97636000	-1.29558000	-2.95260100
H	2.79728000	-1.98894600	-1.35630600
H	1.22681200	-2.89962300	-3.03781800
H	0.54046900	-1.31192900	-3.29057700
H	-1.29558000	-2.97636000	-2.95260100
H	-1.98894600	-2.79728000	-1.35630600
H	-1.31192900	-0.54046900	-3.29057700
H	-2.89962300	-1.22681200	-3.03781800
H	-2.79728000	1.98894600	-1.35630600
H	-2.97636000	1.29558000	-2.95260100
H	-0.54046900	1.31192900	-3.29057700
H	-1.22681200	2.89962300	-3.03781800
C	4.40963200	2.27160600	0.47850400
H	5.32359100	2.14885800	-0.09067800
C	2.27160600	-4.40963200	0.47850400
H	2.14885800	-5.32359100	-0.09067800
C	-4.40963200	-2.27160600	0.47850400
H	-5.32359100	-2.14885800	-0.09067800
C	-2.27160600	4.40963200	0.47850400
H	-2.14885800	5.32359100	-0.09067800
C	-4.27168200	-3.25172100	1.43295600
H	-5.07177400	-3.94504900	1.66173700
C	-3.04828100	-3.33314300	2.09792200
H	-2.86350000	-4.08301200	2.85896500
C	3.25172100	-4.27168200	1.43295600
H	3.94504900	-5.07177400	1.66173700
C	3.33314300	-3.04828100	2.09792200
H	4.08301200	-2.86350000	2.85896500
C	3.04828100	3.33314300	2.09792200
H	2.86350000	4.08301200	2.85896500
C	4.27168200	3.25172100	1.43295600
H	5.07177400	3.94504900	1.66173700
C	-3.33314300	3.04828100	2.09792200
H	-4.08301200	2.86350000	2.85896500
C	-3.25172100	4.27168200	1.43295600
H	-3.94504900	5.07177400	1.66173700

Pb(MeDO2PA) TSAP -1754.961465 Hartree

Pb	0.00000000	-0.00000000	0.07741292
O	-1.55624000	-0.78461878	2.01384206
O	1.55624000	0.78461878	2.01384206
N	-0.19545705	2.54215613	0.91580773
N	0.19545705	-2.54215613	0.91580773
N	-0.99087717	1.95411993	-1.70508918
N	1.90134383	0.97738593	-1.69117124
N	0.99087717	-1.95411993	-1.70508918
N	-1.90134383	-0.97738593	-1.69117124
O	-2.05489993	-2.24508765	3.63549919
O	2.05489993	2.24508765	3.63549919
C	0.14728180	2.64298588	-2.32503929
H	0.54573484	3.35485393	-1.59962837
H	-0.18469024	3.23841981	-3.18997432
C	1.25402278	1.70862584	-2.78169327
H	0.85115474	0.98731679	-3.49424819
H	1.99833975	2.30272580	-3.33688735
C	2.67581980	-0.16354412	-2.18752219
H	3.36348976	0.15383082	-2.98844825
H	3.30615684	-0.50907005	-1.36326319
C	-1.84143922	1.31639485	-2.71376409
H	-2.52388424	2.05349682	-3.16497712
H	-1.20294026	0.97041279	-3.52784810
C	-1.77062913	2.88707799	-0.89366022
H	-2.66879912	2.37079502	-0.53862714
H	-2.11773116	3.74234395	-1.49409127
C	-1.01731408	3.39641609	0.30940870
C	-1.18815405	4.69611512	0.77685761
H	-1.84922607	5.38053408	0.25766359
C	-0.49066300	5.09174421	1.91004355
H	-0.60580098	6.09824824	2.29747148
C	0.36582503	4.19281326	2.53093458
H	0.94129107	4.45692232	3.40859553
C	0.49393100	2.91686921	1.99419867
C	1.45333803	1.89486726	2.60737771
C	2.77779886	1.85891798	-0.92397235
H	3.18348190	1.31632405	-0.06902833
H	2.21618888	2.70856301	-0.53538139
H	3.60642184	2.23790593	-1.54201742
C	-0.14728180	-2.64298588	-2.32503929
H	-0.54573484	-3.35485393	-1.59962837
H	0.18469024	-3.23841981	-3.18997432
C	-1.25402278	-1.70862584	-2.78169327
H	-0.85115474	-0.98731679	-3.49424819
H	-1.99833975	-2.30272580	-3.33688735
C	-2.67581980	0.16354412	-2.18752219
H	-3.36348976	-0.15383082	-2.98844825
H	-3.30615684	0.50907005	-1.36326319
C	1.84143922	-1.31639485	-2.71376409
H	2.52388424	-2.05349682	-3.16497712
H	1.20294026	-0.97041279	-3.52784810
C	1.77062913	-2.88707799	-0.89366022
H	2.66879912	-2.37079502	-0.53862714
H	2.11773116	-3.74234395	-1.49409127
C	1.01731408	-3.39641609	0.30940870
C	1.18815405	-4.69611512	0.77685761
H	1.84922607	-5.38053408	0.25766359
C	0.49066300	-5.09174421	1.91004355
H	0.60580098	-6.09824824	2.29747148
C	-0.36582503	-4.19281326	2.53093458
H	-0.94129107	-4.45692232	3.40859553
C	-0.49393100	-2.91686921	1.99419867
C	-1.45333803	-1.89486726	2.60737771
C	-2.77779886	-1.85891798	-0.92397235
H	-3.18348190	-1.31632405	-0.06902833
H	-2.21618888	-2.70856301	-0.53538139
H	-3.60642184	-2.23790593	-1.54201742

Pb(MeDO2PA) SAP -1754.958209 Hartree

Pb	-0.00000000	0.00000000	-0.22665391
N	-1.51303598	1.60806612	1.56383409
N	-1.55896592	-1.48207688	1.59980614
N	1.51303598	-1.60806612	1.56383409
N	1.55896592	1.48207688	1.59980614
N	0.42483706	-2.58999188	-0.92549588
N	-0.42483706	2.58999188	-0.92549588
O	1.43182797	1.02887212	-2.16306095
O	-1.43182797	-1.02887212	-2.16306095
O	2.00809192	2.73485012	-3.49236198
O	-2.00809192	-2.73485012	-3.49236198
C	-1.25067005	3.35117610	-0.20748594
C	1.25067005	-3.35117610	-0.20748594
C	-2.53333796	0.75161811	2.18480512
C	-2.00469492	-0.58768787	2.66933813
C	-0.75359689	-2.58458885	2.13287414
C	0.64070411	-2.19115082	2.59199411
C	2.53333796	-0.75161811	2.18480512
C	2.00469492	0.58768787	2.66933813
C	0.75359689	2.58458885	2.13287414
C	-0.64070411	2.19115082	2.59199411
C	-2.16601302	2.64074410	0.75594108
C	-2.70432692	-2.02677291	0.87558316
C	2.16601302	-2.64074410	0.75594108
C	2.70432692	2.02677291	0.87558316
H	-2.94514402	2.15235407	0.16125210
H	-2.66807102	3.38289810	1.39465908
H	-3.37511490	-2.58633591	1.54602118
H	-2.35413892	-2.69818492	0.09110817
H	2.66807102	-3.38289810	1.39465908
H	2.94514402	-2.15235407	0.16125210
H	2.35413892	2.69818492	0.09110817
H	3.37511490	2.58633591	1.54602118
H	-3.32542097	0.58906409	1.45122413
H	-3.00440395	1.26349512	3.03825112
H	-2.79869190	-1.07213988	3.26087715
H	-1.17176592	-0.42670384	3.35603012
H	-1.26362387	-3.05478085	2.99032616
H	-0.69077589	-3.35014286	1.35895115
H	1.11587013	-3.08579280	3.02418912
H	0.56154911	-1.47485781	3.41107110
H	3.00440395	-1.26349512	3.03825112
H	3.32542097	-0.58906409	1.45122413
H	1.17176592	0.42670384	3.35603012
H	2.79869190	1.07213988	3.26087715
H	0.69077589	3.35014286	1.35895115
H	1.26362387	3.05478085	2.99032616
H	-0.56154911	1.47485781	3.41107110
H	-1.11587013	3.08579280	3.02418912
C	1.29416911	-4.73203885	-0.36872086
H	1.96478113	-5.32997783	0.23740814
C	-1.29416911	4.73203885	-0.36872086
H	-1.96478113	5.32997783	0.23740814
C	-0.39555894	-3.14550491	-1.81744285
C	0.39555894	3.14550491	-1.81744285
C	0.45611611	-5.31952688	-1.30766883
H	0.46633613	-6.39437288	-1.45223882
C	-0.40223092	-4.51809491	-2.04621683
H	-1.08142992	-4.92596993	-2.78335282
C	0.40223092	4.51809491	-2.04621683
H	1.08142992	4.92596993	-2.78335282
C	-0.45611611	5.31952688	-1.30766883
H	-0.46633613	6.39437288	-1.45223882
H	-3.27502895	-1.22965493	0.39821216
H	3.27502895	1.22965493	0.39821216
C	1.36221194	2.22372812	-2.56381297
C	-1.36221194	-2.22372812	-2.56381297

Pb(H2DO2PA) TSAP -1676.426256 Hartree

Pb	0.00000000	0.00000000	0.05746300
O	-2.18738600	1.27117000	1.25152000
O	2.18738600	-1.27117000	1.25152000
N	2.25429500	1.36609300	0.75831700
N	-2.25429500	-1.36609300	0.75831700
N	1.03947000	1.98698100	-1.59620800
N	1.69683300	-0.86995100	-1.63121500
N	-1.03947000	-1.98698100	-1.59620800
N	-1.69683300	0.86995100	-1.63121500
O	-3.54492300	1.02696100	3.02194100
O	3.54492300	-1.02696100	3.02194100
C	2.20789100	1.43402900	-2.29508800
H	3.05663400	1.45219000	-1.60879700
H	2.47910600	2.06056000	-3.15694800
C	1.99463500	0.00558700	-2.75793700
H	1.16717700	-0.04674300	-3.47090900
H	2.89219700	-0.32565800	-3.29852300
C	1.40376900	-2.25693800	-1.97908300
H	2.12101800	-2.65019500	-2.71225300
H	1.51715400	-2.85033600	-1.06779300
C	0.00000000	2.40936500	-2.54095000
H	0.15549000	3.45090300	-2.85517700
H	0.09423500	1.80376500	-3.44467400
C	1.42696700	3.09189300	-0.72212100
H	0.51657200	3.54086100	-0.30935300
H	1.93701300	3.88764000	-1.28687100
C	2.30606500	2.65173000	0.42319700
C	3.12875300	3.54937900	1.09788800
H	3.16890400	4.59002200	0.79606100
C	3.89413800	3.07747700	2.15521300
H	4.54186300	3.75376100	2.70265700
C	3.83414000	1.73148800	2.49553400
H	4.42200800	1.31588100	3.30376100
C	2.99955700	0.89781600	1.76108300
C	2.90406500	-0.60224300	2.04446300
C	-2.20789100	-1.43402900	-2.29508800
H	-3.05663400	-1.45219000	-1.60879700
H	-2.47910600	-2.06056000	-3.15694800
C	-1.99463500	-0.00558700	-2.75793700
H	-1.16717700	0.04674300	-3.47090900
H	-2.89219700	0.32565800	-3.29852300
C	-1.40376900	2.25693800	-1.97908300
H	-2.12101800	2.65019500	-2.71225300
H	-1.51715400	2.85033600	-1.06779300
C	0.00000000	-2.40936500	-2.54095000
H	-0.15549000	-3.45090300	-2.85517700
H	-0.09423500	-1.80376500	-3.44467400
C	-1.42696700	-3.09189300	-0.72212100
H	-0.51657200	-3.54086100	-0.30935300
H	-1.93701300	-3.88764000	-1.28687100
C	-2.30606500	-2.65173000	0.42319700
C	-3.12875300	-3.54937900	1.09788800
H	-3.16890400	-4.59002200	0.79606100
C	-3.89413800	-3.07747700	2.15521300
H	-4.54186300	-3.75376100	2.70265700
C	-3.83414000	-1.73148800	2.49553400
H	-4.42200800	-1.31588100	3.30376100
C	-2.99955700	-0.89781600	1.76108300
C	-2.90406500	0.60224300	2.04446300
H	2.44772400	-0.84799300	-0.94429000
H	-2.44772400	0.84799300	-0.94429000

Pb(H2DO2PA) SAP -1676.42343 Hartree

Pb	0.00000000	0.00000000	0.17811600
N	0.00000000	2.23781000	-1.39603100
N	1.93664700	-0.00450000	-1.55172900
N	0.00000000	-2.23781000	-1.39603100
N	-1.93664700	0.00450000	-1.55172900
N	1.74009300	-2.01653400	0.80999800
N	-1.74009300	2.01653400	0.80999800
O	-2.47296900	-0.53506600	1.33602400
O	2.47296900	0.53506600	1.33602400
O	-3.84482300	0.17827900	2.96297900
O	3.84482300	-0.17827900	2.96297900
C	-1.49511800	3.23602600	0.33800200
C	1.49511800	-3.23602600	0.33800200
C	1.36085200	2.35361200	-1.94162300
C	1.88743600	1.06279900	-2.54169600
C	2.24507800	-1.32333500	-2.10379500
C	0.98606300	-2.09377600	-2.47838900
C	-1.36085200	-2.35361200	-1.94162300
C	-1.88743600	-1.06279900	-2.54169600
C	-2.24507800	1.32333500	-2.10379500
C	-0.98606300	2.09377600	-2.47838900
C	-0.27938900	3.39352600	-0.53966300
C	0.27938900	-3.39352600	-0.53966300
H	0.58685400	3.53522000	0.11634300
H	-0.37742700	4.31167900	-1.13749700
H	0.37742700	-4.31167900	-1.13749700
H	-0.58685400	-3.53522000	0.11634300
H	2.02074100	2.64834900	-1.11979400
H	1.41564900	3.15024400	-2.69834100
H	2.88093300	1.26648400	-2.96433000
H	1.26305400	0.73069400	-3.37537800
H	2.86336000	-1.23650100	-3.00729900
H	2.83713900	-1.87393900	-1.37561600
H	1.27227000	-3.08250000	-2.86660000
H	0.50117400	-1.56860200	-3.30291400
H	-1.41564900	-3.15024400	-2.69834100
H	-2.02074100	-2.64834900	-1.11979400
H	-1.26305400	-0.73069400	-3.37537800
H	-2.88093300	-1.26648400	-2.96433000
H	-2.83713900	1.87393900	-1.37561600
H	-2.86336000	1.23650100	-3.00729900
H	-0.50117400	1.56860200	-3.30291400
H	-1.27227000	3.08250000	-2.86660000
C	2.30796400	-4.31894600	0.65741400
H	2.10467700	-5.29856700	0.24011100
C	-2.30796400	4.31894600	0.65741400
H	-2.10467700	5.29856700	0.24011100
C	2.78704600	-1.79929900	1.60540900
C	-2.78704600	1.79929900	1.60540900
C	3.38354700	-4.11064900	1.51147900
H	4.03355600	-4.93623600	1.78060000
C	3.62887500	-2.83439500	1.99940700
H	4.46176900	-2.62036000	2.65670200
C	-3.62887500	2.83439500	1.99940700
H	-4.46176900	2.62036000	2.65670200
C	-3.38354700	4.11064900	1.51147900
H	-4.03355600	4.93623600	1.78060000
C	-3.05891400	0.35187900	2.01646000
C	3.05891400	-0.35187900	2.01646000
H	2.59917800	0.22998900	-0.81445100
H	-2.59917800	-0.22998900	-0.81445100

Pb(MeDO2Scz)$^{2+}$ TSAP -1598.292898 Hartree

Pb	0.00000000	-0.00000000	0.08630087
O	1.61144209	0.89542695	2.21741491
O	-1.61144209	-0.89542695	2.21741491
N	-0.02347700	2.63856594	0.99328003
N	0.02347700	-2.63856594	0.99328003
N	-0.81899999	2.03919105	-1.56611500
N	-1.96503085	-0.78014100	-1.54288717
N	0.81899999	-2.03919105	-1.56611500
N	1.96503085	0.78014100	-1.54288717
N	0.54549399	2.91535689	2.18800804
H	0.30784695	3.76173985	2.68890710
N	1.92409203	2.34643086	3.93202400
H	2.62353406	1.73768787	4.32242996
H	1.89306099	3.29495584	4.26774206
N	-0.54549399	-2.91535689	2.18800804
H	-0.30784695	-3.76173985	2.68890710
N	-1.92409203	-2.34643086	3.93202400
H	-2.62353406	-1.73768787	4.32242996
H	-1.89306099	-3.29495584	4.26774206
C	-1.70858496	1.47979107	-2.59489203
H	-1.09116995	1.07067515	-3.39522006
H	-2.30947801	2.27518207	-3.05742998
C	-2.64747891	0.41319099	-2.06280009
H	-3.25250992	0.82056591	-1.24846706
H	-3.34947890	0.13368801	-2.86205410
C	-1.41413581	-1.59717091	-2.63077822
H	-0.96409785	-0.93314884	-3.37060118
H	-2.22657979	-2.12808192	-3.14912325
C	-0.39614676	-2.62234089	-2.17052629
H	-0.84529772	-3.28365196	-1.42839932
H	-0.12462873	-3.25184582	-3.03014433
C	1.70858496	-1.47979107	-2.59489203
H	1.09116995	-1.07067515	-3.39522006
H	2.30947801	-2.27518207	-3.05742998
C	2.64747891	-0.41319099	-2.06280009
H	3.25250992	-0.82056591	-1.24846706
H	3.34947890	-0.13368801	-2.86205410
C	1.41413581	1.59717091	-2.63077822
H	0.96409785	0.93314884	-3.37060118
H	2.22657979	2.12808192	-3.14912325
C	0.39614676	2.62234089	-2.17052629
H	0.84529772	3.28365196	-1.42839932
H	0.12462873	3.25184582	-3.03014433
C	-1.52528203	3.06872897	-0.80539794
H	-2.54962701	2.73471791	-0.60598995
H	-1.62406208	3.98919400	-1.40316288
C	-0.90995904	3.42585592	0.51826608
C	-1.40455010	4.66149386	1.19443216
H	-1.74842308	4.44176178	2.21055215
H	-2.23491412	5.10217385	0.64339819
H	-0.60945514	5.41271889	1.26067320
C	1.38940004	1.98300190	2.74669998
C	1.52528203	-3.06872897	-0.80539794
H	2.54962701	-2.73471791	-0.60598995
H	1.62406208	-3.98919400	-1.40316288
C	0.90995904	-3.42585592	0.51826608
C	1.40455010	-4.66149386	1.19443216
H	1.74842308	-4.44176178	2.21055215
H	2.23491412	-5.10217385	0.64339819
H	0.60945514	-5.41271889	1.26067320
C	-1.38940004	-1.98300190	2.74669998
C	-2.90553980	-1.56275210	-0.73531721
H	-3.76661779	-1.88482611	-1.33730422
H	-3.25617483	-0.96049317	0.10335483
H	-2.41085976	-2.44330510	-0.32648526
C	2.90553980	1.56275210	-0.73531721
H	3.25617483	0.96049317	0.10335483
H	2.41085976	2.44330510	-0.32648526
H	3.76661779	1.88482611	-1.33730422

Pb(MeDO2Scz)$^{2+}$ SAP -1598.286266 Hartree

Pb	0.00000000	0.00000000	0.15521697
N	-1.31474601	-1.78778981	-1.49800526
N	-1.71225908	1.25347719	-1.51546795
N	1.31474601	1.78778981	-1.49800526
N	1.71225908	-1.25347719	-1.51546795
N	-0.17727107	-2.64402104	0.94882569
N	0.17727107	2.64402104	0.94882569
N	-0.51779124	3.05683284	2.03603128
N	0.51779124	-3.05683284	2.03603128
O	-1.53351420	1.02153779	2.24514103
O	1.53351420	-1.02153779	2.24514103
C	-2.46117701	-1.06801177	-2.08636922
C	-2.14021803	0.33257029	-2.57515006
C	-1.03770909	2.43321626	-2.07715881
C	0.37400293	2.15906535	-2.55501979
C	2.46117701	1.06801177	-2.08636922
C	2.14021803	-0.33257029	-2.57515006
C	1.03770909	-2.43321626	-2.07715881
C	-0.37400293	-2.15906535	-2.55501979
C	-1.80232901	-2.97192989	-0.78499740
C	-2.86497612	1.68561908	-0.72126794
C	1.80232901	2.97192989	-0.78499740
C	2.86497612	-1.68561908	-0.72126794
H	-2.82151702	-2.77781495	-0.43148341
H	-1.89130296	-3.82452982	-1.47520549
H	-3.60215111	2.20568213	-1.34918391
H	-2.53127116	2.36019700	0.06667414
H	1.89130296	3.82452982	-1.47520549
H	2.82151702	2.77781495	-0.43148341
H	2.53127116	-2.36019700	0.06667414
H	3.60215111	-2.20568213	-1.34918391
H	-3.24257004	-1.01593187	-1.32602424
H	-2.88094797	-1.63451569	-2.93033529
H	-3.03876202	0.72716632	-3.07253705
H	-1.36551000	0.29673539	-3.34198104
H	-1.61570607	2.83438934	-2.92304778
H	-1.02824513	3.20618018	-1.30795472
H	0.73959493	3.05224941	-3.08151368
H	0.35763697	1.36200742	-3.29853887
H	2.88094797	1.63451569	-2.93033529
H	3.24257004	1.01593187	-1.32602424
H	1.36551000	-0.29673539	-3.34198104
H	3.03876202	-0.72716632	-3.07253705
H	1.02824513	-3.20618018	-1.30795472
H	1.61570607	-2.83438934	-2.92304778
H	-0.35763697	-1.36200742	-3.29853887
H	-0.73959493	-3.05224941	-3.08151368
H	-3.34996111	0.83097301	-0.24871505
H	3.34996111	-0.83097301	-0.24871505
H	-0.31939127	3.95448380	2.45833438
H	0.31939127	-3.95448380	2.45833438
C	-1.03254704	-3.43137900	0.42185258
C	-1.38113102	-4.78260707	0.95506142
H	-2.23160099	-5.20543703	0.42093035
H	-1.63833906	-4.73318418	2.01819542
H	-0.53739100	-5.47244203	0.84042338
C	1.03254704	3.43137900	0.42185258
C	1.38113102	4.78260707	0.95506142
H	2.23160099	5.20543703	0.42093035
H	1.63833906	4.73318418	2.01819542
H	0.53739100	5.47244203	0.84042338
C	-1.35393624	2.16417975	2.66490716
C	1.35393624	-2.16417975	2.66490716
N	1.99150184	-2.64947528	3.74869776
H	2.52539781	-2.00007133	4.30115584
H	1.75906485	-3.53677033	4.16340865
N	-1.99150184	2.64947528	3.74869776
H	-2.52539781	2.00007133	4.30115584
H	-1.75906485	3.53677033	4.16340865

$Pb(CRYPT)^{2+}$ -1612.28944763 Hartree

Pb	0.51049700	0.09303000	-0.29029200
O	-0.40414000	1.20468500	1.88327000
O	0.78039800	-1.23349600	1.87116000
O	3.07256400	-0.55153000	-0.90155700
O	2.48934000	1.81225300	0.33244600
O	-2.11453500	0.77505100	-1.17731200
O	-1.57156700	-1.65387800	-0.45995200
N	-0.24976800	2.79019100	-0.38342500
N	1.17655400	-2.64328300	-0.51307200
C	-0.97550100	3.24428100	0.81382600
H	-1.80778000	3.90681600	0.54773500
H	-0.29916400	3.84270200	1.42488100
C	-1.48821100	2.10726600	1.67185100
H	-1.81970100	2.50820600	2.63516300
H	-2.32839500	1.56804300	1.22288000
C	-0.58536900	0.32727400	2.98849000
H	-1.47141700	-0.29858500	2.82877700
H	-0.71667900	0.90932400	3.90635700
C	0.65157400	-0.51562000	3.09539700
H	1.53732800	0.10883000	3.25782400
H	0.54940500	-1.22218200	3.92476300
C	1.88093100	-2.14202200	1.82609100
H	1.99984400	-2.61723900	2.80431200
H	2.79793200	-1.58783300	1.60229400
C	1.55635900	-3.20019700	0.79552800
H	2.40859700	-3.88412200	0.69760600
H	0.72610100	-3.79332400	1.18150900
C	2.27363300	-2.71083600	-1.48561200
H	1.89902800	-2.33590000	-2.44206000
H	2.59578600	-3.75150300	-1.64200600
H	4.99582400	0.18363900	-1.11336600
H	4.41929300	0.00401600	0.56391900
C	3.64366700	1.70574300	-0.49579300
H	4.43084000	2.36507400	-0.11546800
H	3.40505800	1.99827100	-1.52519100
C	2.03344500	3.15537300	0.49430800
H	2.88546100	3.83921100	0.44033000
H	1.62309400	3.21169800	1.50370000
C	1.00315900	3.53783500	-0.54739500
H	0.82336500	4.62177500	-0.49411400
H	1.39670700	3.33148400	-1.54683000
C	0.00741300	-3.33261700	-1.06874700
H	0.15893200	-4.42228700	-1.07809900
H	-0.11065100	-3.01287900	-2.10707700
C	-1.28076300	-3.04876900	-0.34125200
H	-2.07542900	-3.62502200	-0.82216000
H	-1.24427700	-3.31965500	0.71795200
C	-2.87256400	-1.26617500	-0.30626300
C	-3.86666500	-2.06783300	0.23096100
H	-3.63280900	-3.06178300	0.58993100
C	-5.17503000	-1.59405200	0.31307800
H	-5.94493700	-2.23096100	0.73256500
C	-5.48244700	-0.32502800	-0.14260200
H	-6.49965400	0.04584900	-0.09647100
C	-4.48261400	0.49415200	-0.66543700
H	-4.73970800	1.48153800	-1.02517400
C	-3.17519500	0.03632600	-0.73282400
C	-2.36382500	2.13950300	-1.51267600
H	-3.00232900	2.59147400	-0.74890100
H	-2.88734300	2.19499100	-2.47149300
C	-1.04990500	2.87120000	-1.60941400
H	-0.45502300	2.46093100	-2.43112800
H	-1.28149700	3.91664700	-1.86416200
C	3.47701100	-1.89940700	-1.08564400
H	3.94187600	-2.27724700	-0.16674500
H	4.22668000	-1.95418500	-1.88311700
C	4.12604300	0.28738300	-0.45486400
